# Characterization of the Polycomb-Group Mark H3K27me3 in Unicellular Algae

**DOI:** 10.3389/fpls.2017.00607

**Published:** 2017-04-26

**Authors:** Pawel Mikulski, Olga Komarynets, Fabio Fachinelli, Andreas P.M. Weber, Daniel Schubert

**Affiliations:** ^1^Institute of Biology, Free University of BerlinBerlin, Germany; ^2^Institute of Genetics, Heinrich-Heine-Universität DüsseldorfDüsseldorf, Germany; ^3^Faculty of Medicine, University of GenevaGeneva, Switzerland; ^4^Institute of Plant Biochemistry, Heinrich-Heine-Universität DüsseldorfDüsseldorf, Germany

**Keywords:** Polycomb, PcG, H3K27me3, *Cyanidioschyzon merolae*, algae, intein, protein splicing, telomere

## Abstract

Polycomb Group (PcG) proteins mediate chromatin repression in plants and animals by catalyzing H3K27 methylation and H2AK118/119 mono-ubiquitination through the activity of the Polycomb repressive complex 2 (PRC2) and PRC1, respectively. PcG proteins were extensively studied in higher plants, but their function and target genes in unicellular branches of the green lineage remain largely unknown. To shed light on PcG function and *modus operandi* in a broad evolutionary context, we demonstrate phylogenetic relationship of core PRC1 and PRC2 proteins and H3K27me3 biochemical presence in several unicellular algae of different phylogenetic subclades. We focus then on one of the species, the model red alga *Cyanidioschizon merolae*, and show that H3K27me3 occupies both, genes and repetitive elements, and mediates the strength of repression depending on the differential occupancy over gene bodies. Furthermore, we report that H3K27me3 in *C. merolae* is enriched in telomeric and subtelomeric regions of the chromosomes and has unique preferential binding toward intein-containing genes involved in protein splicing. Thus, our study gives important insight for Polycomb-mediated repression in lower eukaryotes, uncovering a previously unknown link between H3K27me3 targets and protein splicing.

## Introduction

In the eukaryotic cells, transcription state is dependent on the underlying chromatin state. The fundamental structure of chromatin is based on a nucleosome, a complex of 147bp-long fragments of DNA wrapped around the core histone proteins (H2A, H2B, H3, H4). Chromatin state can be influenced by post-translational modifications deposited on the histones, either by direct structural changes in the nucleosomes or recruitment/displacement of secondary proteins involved in chromatin remodeling or transcription. The presence of particular histone modifications often determines the type of the chromatin and correlates with transcriptional activity of the target DNA. Among those, tri-methylation of lysine 27 on histone H3 (H3K27me3) and mono-ubiquitination of histone H2AK118/H2AK119 (H2AK118ub/H2AK119ub) are commonly associated with transcriptionally silent facultative heterochromatin.

Deposition of H3K27me3 and H2AK118ub/H2AK119ub is mediated by Polycomb group (PcG) proteins, whose function was initially shown to control developmentally regulated processes and maintain cell identity (Papp and Müller, 2006; Schuettengruber et al., 2007) in both, animals [reviewed in Schwartz and Pirrotta (2008)] and plants [reviewed in Köhler and Villar (2008)]. PcG proteins form distinct complexes, like Polycomb Repressive Complex 2 (PRC2), involved in H3K27 trimethylation [in metazoans Pcl-PRC2 complex (Nekrasov et al., 2007; Müller and Verrijzer, 2009)], and Polycomb Repressive Complex 1 (PRC1), responsible for H2A mono-ubiquitination. PRC2 in *Drosophila melanogaster*, where it was initially discovered, consists of four core subunits: Enhancer of zeste [E(z)], a catalytic component needed for methylation of H3K27; Extra Sex Combs (ESC), a WD40 motif-containing protein that scaffolds interactions within the complex; Suppressor of zeste (Su(z)12), a Zinc Finger subunit essential for binding of PRC2 to nucleosomes and p55, a nucleosome remodeling factor (Schwartz and Pirrotta, 2013). In turn, PRC1 is formed by: dRING/Sex Combs Extra (Sce); Posterior Sex Combs (Psc), both responsible for mono-ubiquitination activity; Polyhomeotic (Ph), essential for maintaining protein-protein interactions; and Polycomb (Pc), involved in recruitment of the complex to chromatin; and Sex comb on midleg (Scm), important for spreading of PcG silencing (Schwartz and Pirrotta, 2013). Both complexes are functionally connected with each other. In the canonical hierarchical model, initially introduced H3K27me3 mark is recognized by PRC1 and followed by H2A mono-ubiquitination to maintain the repression. However, it was shown that the mechanism of PcG-mediated repression can happen as well in the opposite order, with PRC1 introducing its modification prior to PRC2 activity (Yang et al., 2013; Del Prete et al., 2015).

Polycomb-group complexes were identified also in the plant kingdom. Core PRC1 complex in model flowering plant *Arabidopsis thaliana* consist of AtRING1a/b (equivalent to dRING/Sce), AtBMI1a/b/c and EMF1 (equivalent to Psc), and LHP1 (equivalent to Pc). Although a wide range of PRC1-associated proteins exists (Chen et al., 2016), it is unclear how the combinations of different subunits depend on specific temporal and spatial conditions. In contrast to PRC1, composition of the *Arabidopsis* PRC2 complex is better characterized. *Arabidopsis* PRC2 subunits underwent gene duplication, resulting in the presence of different, partially redundant or independently acting PRC2 complexes (Derkacheva and Hennig, 2014). In the *Arabidopsis* genome the following PRC2 components can be identified: three E(z)-homologs – CURLY LEAF (CLF), SWINGER (SWN), MEDEA (MEA); three Su(z)12-homologs – EMBRYONIC FLOWER 2 (EMF2), VERNALIZATION 2 (VRN2), FERTILIZATION INDEPENDENT SEED 2 (FIS2); as well as single gene subunits: ESC-homolog – FERTILIZATION INDEPENDENT ENDOSPERM (FIE) and p55-homologs – MULTICOPY SUPPRESSOR OF IRA 1-5 (MSI1-5).

One of the aspects of PRC2 biology concerns its origins and the abundance in lower eukaryotes. Due to the absence of PRC2 in the model unicellular species *Saccharomyces cerevisiae* and *S. pombe* (Lachner et al., 2004; Veerappan et al., 2008), the PRC2 appearance was previously thought to co-occur with the emergence of multicellularity. However, high conservation of PRC2 subunits in both, animal and plant lineages implies that PcG genes appeared already in the last common unicellular ancestor (Bowman et al., 2007), before those two kingdoms diverged. In an elegant study, Shaver *et al* (Shaver et al., 2010) identified the novel computational PRC2-homologs in several unicellular species and showed that E(z)-homolog in unicellular green algae *Chlamydomonas reinhardtii* is responsible for mono- and di-methylation of lysine 27 on histone H3. As the tri-methylation activity of *Chlamydomonas* E(z) homolog was not found, this study highlights important differences in the functional conservation of PRC2 components. For instance, although H3K27 tri-methylation is considered to be a prominent histone mark in PRC2-mediated repression, there is a range of H3K27 methylation levels catalyzed by PRC2 that varies between plant and *Drosophila* or mammals. In *Arabidopsis*, PRC2 controls H3K27 tri-methylation and H3K27 di-methylation in the euchromatin (Bastow et al., 2004; Lindroth et al., 2004; Sung et al., 2006), with mono-methylation being catalyzed by ATXR5 and ATXR6 (Jacob and Michaels, 2009; Jacob et al., 2009). However, in metazoans homologs of ATXR5/6 were not found and their PRC2 complexes mediate H3K27 methylation in all contexts (Müller et al., 2002; Ebert et al., 2004; Ferrari et al., 2014). Another difference between species is the type of DNA elements targeted by PRC2. *Drosophila* PRC2 targets both, genes and transposable elements/repetitive sequences (Yin et al., 2011), whereas in *Arabidopsis* the H3K27me3 mark is excluded from the majority of transposable elements/repetitive sequences (Lafos et al., 2011; Deleris et al., 2012; Park et al., 2012).

Even though PRC2-mediated repression has been extensively studied in higher plants and animals, its characterization in lower eukaryotes gathered much less attention. The existence of H3K27me3 in lower eukaryotes is largely unknown (Shaver et al., 2010) and distribution of the mark in the genome and its function were reported only for a handful of species, such as *Neurospora crassa*, *Phaeodactylum tricornutum*, or *Tetrahymena thermophila*. In *N. crassa* H3K27me3 is arranged into broad domains covering 774 genes connected with a full spectrum of functions (Jamieson et al., 2013). In *T. thermophila*, the mark associates with the developmentally regulated genome rearrangements as it occupies sequences eliminated during differentiation of the macronucleus (Liu et al., 2007). In *P. tricornutum*, H3K27me3 covers transposable elements and genes, and its targets are involved in, i.e., signal transduction, development and cell cycle control (Veluchamy et al., 2015).

Given the distinct degrees of phylogenetic conservation of PRC2 and PRC1, and poor characterization of H3K27me3 in lower eukaryotes, we sought to investigate the presence of Polycomb-group homologs and H3K27me3 abundance in several representative unicellular, photosynthetically active eukaryotes. Our scope included the representatives of red algae, green algae and *Glaucophyta* that vary in genome size, genome architecture, ecological niche and metabolism (Matsuzaki et al., 2004; Palenik et al., 2007; Worden et al., 2009; Merchant et al., 2010; Price et al., 2012). After an initial small-scale screen on H3K27me3 presence, we focused on one of the species, *Cyanidioschyzon merolae*, to study genome-wide occupation of H3K27me3 and characterize its targets. *C. merolae* is a unicellular red alga, living in highly acidic environment with high temperatures. It contains a small (16 Mb) genome which was fully sequenced as the first algal genome (Matsuzaki et al., 2004) and assembled as first 100% complete eukaryotic genome (Nozaki et al., 2007). Interestingly, the genome shows extremely simplified structure that contains almost exclusively intron-lacking genes (only 26 genes contain an intron), very low percentage (0,7%) of transposable elements and a novel class of a repetitive element, corresponding to the truncated ORF from White spot syndrome virus (WSV repeat) (Nozaki et al., 2007). Given its unicellularity, evolutionary ancestry, primitive architecture of the genome and availability of sequencing data, we argue that *C. merolae* is a suitable model for studying chromatin repression in an evolutionary context.

Our results confirmed the high conservation of PRC2 core members in lower eukaryotes and the widespread presence of H3K27me3 modification. We report that in *C. merolae* H3K27me3 mark targets both, genes and repetitive elements and is anti-correlated with transcriptional activity. We show that H3K27me3 distribution over its targets is not uniform, but can be grouped into several different clusters that correlate with different levels of repression. Furthermore, we demonstrate that H3K27me3 has a preferential chromosomal localization toward telomeric and subtelomeric regions. For the first time, we also reveal that H3K27me3 target genes are enriched in the functional class of intein-mediated protein splicing. Moreover, by deposition of RNA- and ChIP-sequencing data, we provide a resource for studies on the chromatin and transcriptome in the model red alga *C. merolae*.

## Materials and Methods

### Phylogenetic Analysis and Homology Search

Sequences of PRC1 and PRC2 components from *A. thaliana* and *D. melanogaster* were selected from TAIR10 and NCBI databases, respectively, and used as the queries. Homology searches were done using BLAST against organism-specific databases at: https://phytozome.jgi.doe.gov/pz/portal.html (*C. reinhardtii* v5.5 and *Micromonas pusilla* CCMP1545 v3.0), http://merolae.biol.s.u-tokyo.ac.jp/ (*C. merolae*), http://genome.jgi.doe.gov/Ostta4/Ostta4.home.html (*Ostreococcus tauri* v2.0), http://cyanophora.rutgers.edu/cyanophora/home.php (*Cyanophora paradoxa*).

The highest scoring candidate proteins were used for BLAST searches against the TAIR10 genome annotation to confirm a reciprocal match to the protein used as an initial query. The sequences were aligned using ClustalXv2.0 (Larkin et al., 2007) and conserved blocks were selected by gBlocks v0.91b (Talavera and Castresana, 2007). For substitution model estimation, ProtTestv3.4 was employed (Abascal et al., 2005). Phylogenetic trees were created using a Bayesian approach in Mr Bayesv3.2 (Ronquist et al., 2012), self-compiled developer version r1067 with implemented LG model. The runs were done with 20 mln generations. Sequences from *D. melanogaster* were selected as an out group. Consensus trees and alignments were visualized with Figtree v1.4.2 (Rambaut, 2009) and Jalviewv2 (Waterhouse et al., 2009), respectively.

### Algae Growth

*Cyanidioschyzon merolae* cells were grown at 42°C with shaking at 150 rpm. The cultures were kept under continuous light, with light intensity of 70 μmol.

### Western Blot

Around 50.000 algae cells were diluted in Laemmli’s sample buffer, and 3–7 μl of total protein/lane was subjected to sodium dodecyl sulfate-polyacrylamide gel electrophoresis (SDS-PAGE) using 12% SDS-polyacrylamide gel. The resolved proteins were transferred onto polyvinylidenedifluoride (PVDF) membranes (Immobilon-P membrane, Milipore) using a Vertical Electrophoresis Cell (Bio-Rad). Following transfer, membranes were washed with phosphate buffered saline containing 0.05% Tween-20 (PBST) and then blocked in 5% milk diluted in PBST for 1 h at room temperature. The membranes were then incubated overnight with anti_H3K27me3 (N 07-449, Millipore or C15410195, Diagenode) and anti H3 (ab1791-100, Abcam) rabbit polyclonal antibodies 1:2000 in PBST containing 5% milk at 4°C. After washing three times for 10 min in PBST, membranes were incubated with peroxidase-labeled secondary antibody for 1 h at room temperature. The membranes were washed three times for 10 min in PBST, incubated with SuperSignal West Femto Chemiluminescence Substrate (Thermo Scientific) for 1 min, and exposed for 1–2 min. Commercial histone extract from calf thymus (Sigma, #9064-47-5) was used as a positive control for H3K27me3 detection.

### Competition Assay

Pre-incubation of anti-H3K27me3 antibody (C15410195, Diagenode) and H3K27me3 peptide (Intavis) was done for 2 h in RT in PBST, with occasional mixing. The peptide-to-antibody molar ratio was 50:1. A control solution – PBST with just antibody at 2X final concentration – was used at the same time. At the end of the pre-incubation, 4% BSA blocking solution was added to the peptide/antibody mixture to a final concentration of 3% BSA, mixed briefly and added to the membranes. After washing three times for 10 min in PBST, membranes were incubated with peroxidase-labeled secondary antibody for 1 h at room temperature. The membranes were washed three times for 10 min in PBST, incubated with SuperSignal West Femto Chemiluminescence Substrate (Thermo Scientific) for 1 min, and exposed for 1–2 min.

### Chromatin Immunoprecipitation

*Cyanidioschyzon merolae* cells were grown until late log-phase. 40 ml samples were fixed [Formaldehyde, 1% (v/v)] for 10 min, until addition of glycine (to final concentration of 125 mM, 5 min incubation). Superfluous formaldehyde was removed by three washes with ice cold PBS buffer and the remaining cell pellet was resuspended in 4 ml of Extraction Buffer (50 mM Tris-HCl pH 8, 10 mM EDTA, 1% SDS) with Protease Inhibitor Cocktail (Roche). The samples were sonicated for 5 min with 30 s ON/30 s OFF cycle using Bioruptor Plus device (Diagenode) and cleared by two rounds of centrifugation (13000 rpm, 4°C, 10 min). Subsequent steps were performed as in the Plant ChIP-seq kit protocol (Diagenode) with higher volume of sample taken aside as an input (1:5 of chromatin for IP). Immunoprecipitation was done using anti-H3K27me3 Polyclonal Premium antibody (C15410195, Diagenode) and, as a negative control, IgG fraction from rabbit (C15410206, Diagenode). Quality and fragment size of immunoprecipitated DNA and input samples were measured using agarose gel electrophoresis and Bioanalyzer 2100 (Agilent Technologies).

### Real Time PCR

DNA samples obtained from ChIP were used for H3K27me3 enrichment analysis for several target genes by real-time quantitative PCR. Reactions were prepared using KAPA SYBR FAST qPCR Mastermix (KapaBiosystems), according to the manufacturer’s protocol, and run on an iQ5 detection system (Biorad) using a 2-step program. Differences in H3K27me3 enrichment on target genes were scored by comparison of % recovery of input and standard error values. 5′- > 3′ sequences of oligonucleotides used for amplification were as follows: CmMADS- forward: GGATGAGAAAGCGAGAAATACGA and reverse: TCACAATGCCGATCTCACAG; CmEIF-4A – forward: TGTACGATATGATCCAGAGAAGAG and reverse: TGTAGATTTGCTCCTTGAAACC; Cm60S – forward: AAGTTTCGCTGTACGCTTGG and reverse: TAACCAGGACCATATCGCCG.

### ChIP-seq

DNA library was prepared with MicroPlex Library Preparation Kit (Diagenode) and sequenced on HiSeq 2000 sequencer (Illumina Inc.). Quality of paired-end raw reads was assessed using FastQC v0.11.4. The reads were trimmed according to the quality and mapped to the reference in Bowtie v2.2.6, with standard options. Peaks were called using MACS v2.1 with exclusion of clonal reads. The new annotation from the *C. merolae* Genome Project v3^1^ was employed as a reference. The correlation between ChIP-seq replicates was scored using Pearson correlation and shown in Supplementary Figure S3A. The data was deposited in the Gene Expression Omnibus under the series GSE93913.

### RNA Extraction and RNA-seq

*Cyanidioschyzon merolae* cells were grown until late log-phase. RNA was isolated using RNeasy Plant Mini Kit (Qiagen), following a standard protocol. cDNA synthesis and DNase treatment were performed using RevertAid First Strand cDNA Synthesis Kit (Thermo Scientific) with oligo(dT) primers, according to a standard manual. The quality and concentration of samples were measured spectrometrically using Nanodrop 1000 (Thermo Scientific) and electrophoretically using Bioanalyzer 2100 (Agilent Technologies). Libraries were constructed using TruSeq RNA Library Prep kit (Illumina) with gel-free library purification based on Agencourt AMPure XP beads (Beckman Coulter, Inc.). The samples were sequenced on HiSeq 2000 device (Illumina Inc.). Paired-end raw sequencing reads were analyzed using Galaxy implementations (usegalaxy.org) of FastQC program and Tuxedo protocol (Trapnell et al., 2012). The new annotation from *C. merolae* Genome Project v3 was employed as a reference. The correlation between replicates in RNA-seq experiment was scored using Pearson correlation and shown in Supplementary Figure S4. The data was deposited in the Gene Expression Omnibus, under the series GSE93912.

### Bioinformatic Secondary Analysis of NGS Data

Identified H3K27me3 peaks were annotated by intersection with the reference using Bedtools v2.17 (Quinlan and Hall, 2010). Sequences from unannotated peaks were translated *in silico* to ORF in HMMER2GO v0.17 software and the longest ORF was used for homology searches by BLASTP/Pfam against respective protein databases. Alternatively, unannotated peak sequences were used for homology searches by BLASTN against NCBI nucleotide databases: nr/nt and ref_seq. Homology searches were performed in BLAST+ standalone package v2.2.28+ (Camacho et al., 2009). Alignment threshold was set as follows: >50% alignment length and <0.5 *E*-value. Complete genome records in nucleotide databases were used to form negative GI list and excluded from the BLASTN searches. Only the top alignment hit was included in the further analyses. Distance between peak and annotated genomic feature was obtained using ‘closest’ command from Bedtoolsv2.17. Mapping of the reads was visualized in IGV v2.3 (Thorvaldsdóttir et al., 2013). Clustering of H3K27me3 enrichment was done on deepTools2 (Ramirez et al., 2016). Gene ontology was inferred by using Singular Enrichment Analysis on the AgriGO server (Du et al., 2010) against complete GO list. Protein annotations were extracted from UniProtKB database and used as reference. To select significantly enriched GO terms, Fisher test with Yekutieli adjustment was used a statistical method. *P*-value was set to 0.05 and minimal number of entries kept at 5.

## Results

### Conservation of PRC1 and PRC2 Homologs

In order to assess evolutionary conservation of Polycomb complexes, we performed phylogenetic analysis in the species from various groups of lower plants. We focused on the representatives from subclades: Chlorophyta (green algae), Rhodophyta (red algae), Glaucophyta and Embryophyta. For comparison, a representative from Metazoa, *D. melanogaster*, was included as well. Using reciprocal BLAST searches with full length proteins from *A. thaliana* or *D. melanogaster*, we identified homologs of PRC1 and PRC2 members in several species (**Table 1**).

**Table 1:** Number of identified homologs of PRC2 and PRC1 components in selected species.

Clade		E(z)	ESC	Suz12	P55	BMI1/Psc	RING1A/Sce	LHP1/Pc	EMF1	Scm	H3
Metazoa	*D. melanogaster*	1	1	1	1	1	1	1	0	1	∼100
Embryophyta	*A. thaliana*	3	1	3	6	3	2	1	1	0	15
Chlorophyta	*C. reinhardtii*	1	2	0	2	1	1	0	0	0	35
Chlorophyta	*M. pusilla*	1	1	1	2	0	1	0	0	0	5
Chlorophyta	*O. tauri*	1	1	1	2	0	0	0	0	0	4
Rhodophyta	*C. merolae*	1	1	1	2	0	0	0	0	0	4
Glaucophyta	*C. paradoxa*	0	0	0	2	0	1	0	0	0	12


We found a general absence of PRC1 components: Psc/BMI1, Pc/LHP1, EMF1 and Scm in the algal genomes analyzed, with an exception of Psc/BMI1 homolog in *C. reinhardtii*. In contrast, the RING1 subunit is widely conserved, suggesting a mono-ubiquitylation activity unrelated to PRC1 in algae and protein moonlighting (Jeffery, 2003). In general, an abundance of RING1 homologs in chlorophytes, but the absence of other PRC1 components in lower branches of Archaeplastida used here is consistent with the previous studies (Hennig and Derkacheva, 2009; Berke and Snel, 2015; Chen et al., 2016) and agrees with the notion of lower PRC1 conservation and potential loss of the complex in several phylogenetic branches.

Homology searches on PRC2 revealed broad distribution of complex components: E(z), ESC and p55, consistently with results published elsewhere (Shaver et al., 2010; Butenko and Ohad, 2011; Kim et al., 2013). Importantly, p55 in higher eukaryotes has various functions and participates also in other complexes than PRC2 (i.e., chromatin assembly factor 1 [CAF1)]. Therefore it still remains to be proven whether a role of p55 homologs is Polycomb-related in lower plants. Moreover, we observed an absence of Suz12 from *C. reinhardtii* and *C. paradoxa*. Similarly to the other studies (Shaver et al., 2010), the lower abundance of this subunit shows that Suz12 was lost in several species, despite the presence of the other PRC2 components. Interestingly, we did not detect any core PRC2 members in the *Cyanophora* representative. Given that E(z) and ESC homologs were present in the representatives of Rhodophyta and Chlorophyta, it suggests the existence of PRC2 in the common algal ancestor and subsequent loss of the complex from *Cyanophora*. However, we could not exclude technical issues coming from incomplete annotation and incorrectly predicated protein models in the *Cyanophora* genome. In addition, we identified mostly one homolog per complex component in all screened species, apart from *Arabidopsis*, which adds another piece of evidence to the frequent gene duplication occurring in flowering plants.

Overall, our results agree with the ancient presence of core PRC2 components and the frequent losses of the Suz12 subunit and PRC1 complex members.

### Phylogenetic Relationship of PRC2 Homologs

**FIGURE 1:**
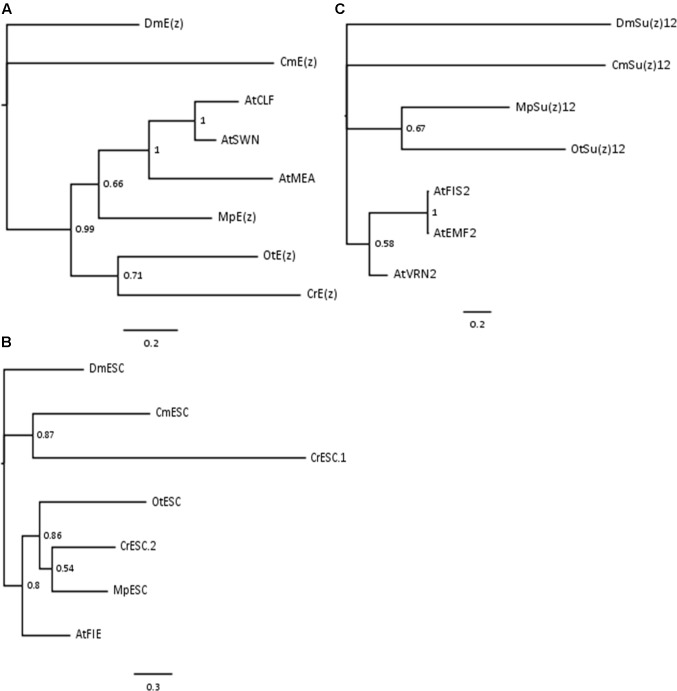
**Bayesian-inferred phylogenetic trees on conserved blocks from homologs of core PRC2 components.** Consensus trees were generated by MrBayes v2 and visualized in FigTree v1.4. Clade credibility values are depicted at the nodes. Branch length corresponds to the rate of expected mutations per site in the protein sequence, according to relevant scale bar below each tree. Sequences from *D. melanogaster* were selected as outgroups. Two-letter abbreviations represent species’ names: Dm – *D. melanogaster*; Cm – *C. merolae*; At – *A. thaliana*; Mp – *M. pusilla*; Ot – *O. tauri*; Cr – *C. reinhardtii*. **(A)** Phylogenetic tree on E(z) homologs. Conserved blocks selected from multiple alignment on following homologs (UniProtKB ID or protein/transcript name from resource databases indicated): DmE(z) – P42124; CmE(z) – CMQ156C; AtCLF – AT2G23380; AtSWN – AT4G02020; AtMEA – AT1G02580; MpE(z) – 59369; OtE(z) – 6642; CrE(z) – Cre17.g746247.t1.1. **(B)** Phylogenetic tree on ESC homologs. Homologs included in the analysis: DmESC – Q24338; CmESC – CMK173C; AtFIE – AT3G20740; MpESC – 49065; OtESC – 22117; CrESC.1 – Cre16.g693750.t1.1; CrESC.2 – Cre03.g180050.t1.1. **(C)** Phylogenetic tree on Su(z)12 homologs. Homologs included in the analysis – DmSu(z)12 – Q9NJG9; CmSu(z)12 – CML082C; AtFIS2 – AT2G35670; AtEMF2 – AT5G51230; AtVRN2 – AT4G16845; MpSu(z)12 – 9357; OtSu(z) – 13623.

Phylogenetic analyses on the conserved domains or full sequences of core PRC2 homologs in lower and higher plants with the creation of Neighbor-Joining (NJ) trees revealed grouping into several distinct classes (Shaver et al., 2010; Kim et al., 2013; Huang et al., 2016). In a different approach, we extracted amino acid sequences from the most abundant PRC2 members, aligned them and automatically selected only the conserved blocks in the multiple alignment (see Material and Methods). With such prepared sequences we created Bayesian trees for E(z)z, ESC and Su(z)12 homologs. Consistently with the published data, the homologs formed defined clades. Bayesian trees for E(z) and ESC sequences generally resolved the evolutionary distance between groups (**Figures 1A,B**). In those cases, we found separation of representatives of Rhodophyta and Viridiplantae, with species from Chlorophyta and Embryophyta forming separate subclades within the latter. The exceptions included one of the ESC homologs from *C. reinhardtii* (CrESC.1), which clustered together with the *C. merolae* homolog, rather than the other green algae. In turn, our analysis on Su(z)12 detected distinct clades for the organisms from Embryophyta, Chlorophyta and Rhodophyta, without grouping of *Arabidopsis* and green algae sequences (**Figure 1C**). Moreover, we noted a phylogenetic distance between *C. merolae* and any other species for all the three proteins.

In summary, our results on Bayesian phylogenetic trees on conserved sequence blocks are in agreement with the published NJ trees using the full length (Kim et al., 2013) or domain-only (Shaver et al., 2010) sequences. Given high conservation of domain architecture in core PRC2 components between the subclades (Shaver et al., 2010; Kim et al., 2013), we conclude that PRC2 is widely distributed and already evolved in a common unicellular ancestor.

### Conservation of Histone H3 Sequences in Various Eukaryotic Organisms

As PRC2 has the canonical function to methylate lysine 27 in histone H3, we decided to analyze protein sequence conservation of histone H3. Amino acid sequences of algal histone H3 were retrieved from the databases of respective genome sequencing projects (see materials and methods) or NCBI, using the canonical histone H3.1 from *Arabidopsis* as a query (Okada et al., 2005). The results revealed a presence of multiple H3 genes in all of the species studied (**Table 1**). Noteworthy, we detected an equal amount of histone H3 gene copies as in the published reports for *M. pusilla* and *C. reinhardtii* (Cui et al., 2015). The number of H3 gene copies in *D.* was taken from the published results (McKay et al., 2015).

**FIGURE 2:**
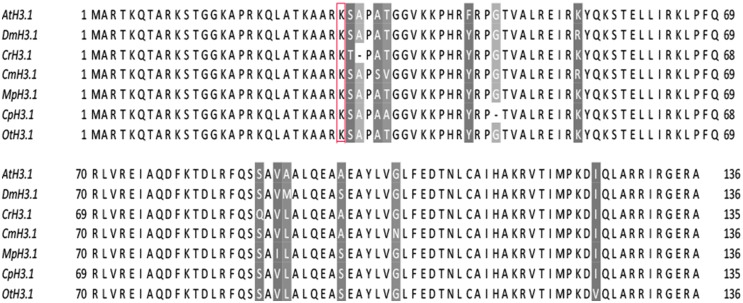
**H3 sequence conservation.** AtH3.1 was used a query for BLAST searches against databases of respective species. The closest homologs were selected and used for multiple alignment in ClustalX, visualized afterward in Jalview v2.9. Color-shading marks non-conserved residues. Red frame correspond to lysine-27, a residue typically modified by PRC2.

Next, the closest H3 homologs between the species were aligned using ClustalX and visualized with Jalview. The alignment revealed overall high amino acid conservation and a presence of lysine-27 in all studied species (**Figure 2**). Moreover, we noted that the sequences around lysine-27, including the underlying motif ARKS, does not show any sequence divergence with an exception of *C. reinhardtii*, which contains threonine-28 instead of serine-28. The results suggested that there is the potential for introduction of the H3K27me3 modification in most of species analyzed. However, in *C. reinhardtii* the presence of threonine-28 next to lysine-27 might not permit detection using commercial antibodies against the methylated H3K27. Moreover, a mass spectrometry analysis on *Chlamydomonas* histone H3 did not reveal the presence of the H3K27me3 modification (Shaver et al., 2010).

### Detection of H3K27me3

**FIGURE 3:**
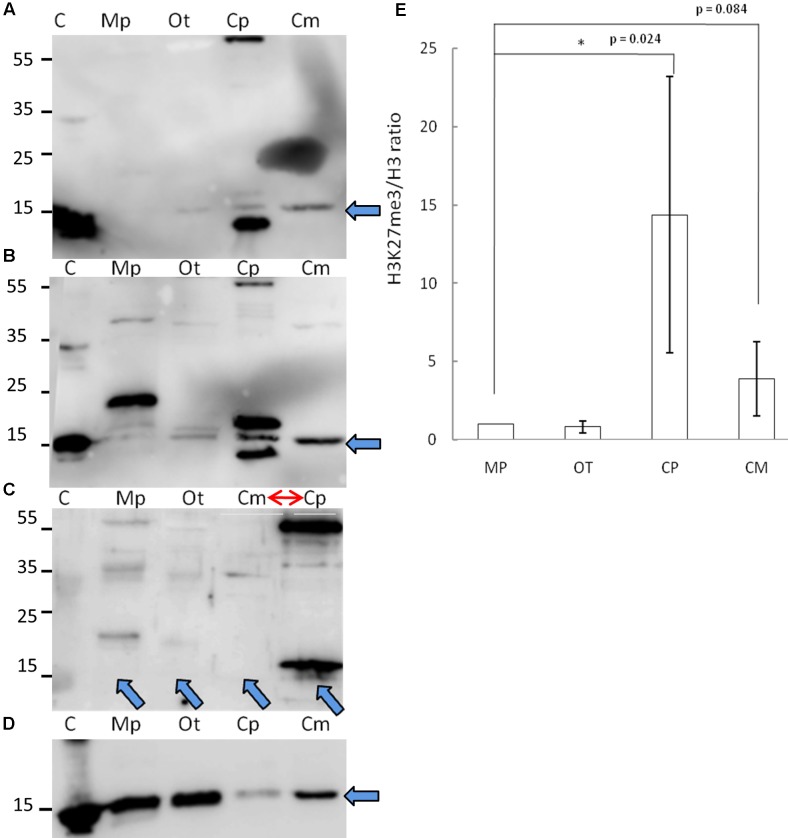
**Biochemical detection of H3K27me3 histone mark.** **(A,B,D)** Western blot analysis of H3K27me3 in different algae (C – histone powder control, Mp – *M. pusilla*, Ot – *O. tauri*, Cp – *C. paradoxa*, Cm – *C. merolae*). Membranes were incubated with antibodies against H3K27me3 [Millipore, N07-449 **(A)** and Diagenode, C15410206 **(B)**] and H3 [Abcam, ab1791-100 **(D)**]. The arrows indicate the band of interest. **(C)** Competition assay done after Western blotting with primary antibodies against H3K27me3 from **(B)**. Note the order change of the samples (red arrow). **(E)** Quantification of Western blotting. Intensity of the bands was obtained using ImageJ. H3K27me3/H3 protein level ratio of three independent experiments is shown. *P*-values were calculated using Student *t*-test. A single asterisk indicates a significance level of *p* < 0.05. Error bars represent standard deviation.

Given the phylogenetic conservation of PRC2 core components and high similarity of amino acid composition of histone H3 in the vast majority of the species, we sought to examine H3K27me3 presence in total protein extracts from selected organisms. Isolated proteins from crude extract were separated on a SDS-PAGE gel and detected using two independently raised anti-H3K27me3 antibodies. Our results revealed the presence of the H3K27me3 modification in *M. pusilla*, *O. tauri*, *C. paradoxa*, and *C. merolae* (**Figures 3A,B**). In order to decipher H3K27me3 relative abundance, we performed western blotting for histone H3 (**Figure 3D**) and calculated H3K27me3/H3 ratios based on band intensity quantification. We detected different relative amounts of the modification in the studied species (**Figure 3E**), with the lowest H3K27me3/H3 ratios for *M. pusilla* and *O. tauri*, intermediate for *C. merolae* and the highest for *C. paradoxa*.

Due to the fact that one of the antibodies detected proteins in the sizes not corresponding to histones, we performed protein competition assay for the confirmation of specific band reactivity. After preincubation with H3K27me3 peptide, we did not detect any band corresponding to this modification for a commercial histone extract from calf thymus (positive control), as well as for *M. pusilla*, *O. tauri*, and *C. merolae* (**Figure 3C**), suggesting a specificity of the antibody. Intensity decrease of the band corresponding to H3K27me3 for *C. paradoxa* was accompanied by the overall signal loss, including the bands of the other molecular weight proteins. Therefore, the presence of H3K27me3 in *C. paradoxa* remains inconclusive.

In summary, we were able to biochemically identify and confirm a presence of H3K27me3 in *M. pusilla*, *O. tauri* and *C. merolae*. This result is consistent with our phylogenetic analyses and suggests that the PRC2 complex is active in these species. For *C. paradoxa*, the biochemical results are unclear: potential absence of H3K27me3 mark would correspond to the lack of PRC2 homologs identified in its genome. Differences in relative H3K27me3 amounts between species imply, e.g., a distinct abundance of genomic targets of H3K27me3 or different H3K27me3 enrichment levels per genomic region. Further work should decipher an impact of such differences in the evolutionary context.

### Characterization of H3K27me3 Target Genes

Based on the broad distribution of PRC2 genes, high sequence conservation of histone H3 and specific signal detected with the anti-H3K27me3 antibody on a protein blot, we selected one of the species, *C. merolae*, to investigate the H3K27me3 abundance further. Firstly, we asked whether the modification has similar targets as in the other organisms. Reciprocal BLAST searches between protein databases of *C. merolae* and *A. thaliana* were used to detect homologous genes of known *A. thaliana* PRC2 targets (Supplementary Figure S1). We selected a MADS box-containing gene CMA095C (CmMADS) as nearly all *Arabidopsis* MADS box genes carry H3K27me3. For negative controls, estimated not to be targeted by H3K27me3, homologous genes for EUKARYOTIC ELONGATION FACTOR EIF4A (CMK028C, CmEIF4A) and 60S RIBOSOMAL PROTEIN L23 (CMS262C, Cm60S) were taken.

**FIGURE 4:**
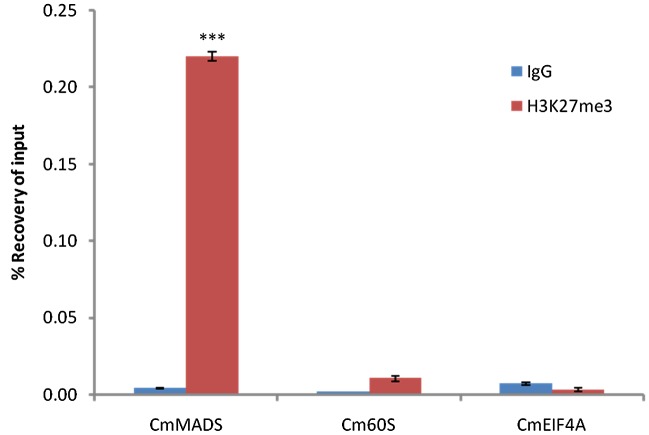
**H3K27me3 abundance on selected genes from *C. merolae*.** Chromatin immunoprecipitation was performed using H3K27me3 antibody (#C15410195, Diagenode) and IgG (#C15410206, Diagenode) for negative control, followed by RT-PCR analysis on a putative H3K27me3 target (CmMADS) and housekeeping genes as the putative non-H3K27me3 targets (Cm60S, CmEIF4A). Selected genes relate to following locus IDs from *C. merolae* genome project: CmMADS – CMA095C, Cm60S – CMS262C, CmEIF4A – CMK028C. Error bars correspond to the standard deviation from three biological replicates. Significance was calculated using Student’s *t*-test for the comparison between CmMADS and either, Cm60S, or CmEIF4A. A triple asterisk indicates the significance level of *p* < 0.001.

Next, we performed Chromatin-immunoprecipitation (ChIP) using anti-H3K27me3 antibody and analyzed the expression of candidate genes by RT-PCR. As a result, we were able to show significant enrichment of H3K27me3 on CmMADS and low abundance of the mark on negative targets: CmEIF4A and Cm60S (**Figure 4**). Overall, we were able to identify positive target of H3K27me3 and show high enrichment of the mark comparing to negative loci.

### H3K27me3 Genome-wide Distribution – Peak Identification

In order to characterize the targets of H3K27me3 and the distribution of H3K27me3 peaks in a genome-wide scale, we created a sequencing library and performed chromatin-immunoprecipitation coupled with sequencing (ChIP-seq) for the samples: H3K27me3-bound DNA, H3-bound DNA (both in triplicate) and input.

**FIGURE 5:**
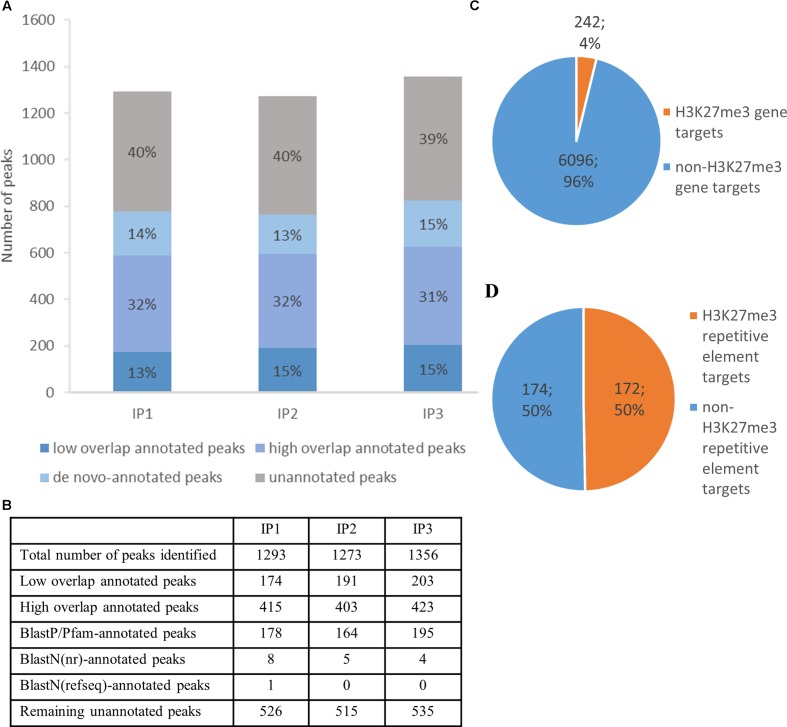
**H3K27me3 genome-wide distribution and peak annotation.** **(A,B)** – Number of peaks identified in three different biological replicates from chromatin immunoprecipitation experiment (IP1-3) in ‘high overlap annotated peaks,’ ‘low overlap annotated peaks’ category, ‘*de novo*-annotated’ and ‘unannotated’ categories (see main text). **(C,D)** – Characterization of H3K27me3 peaks according to genomic element type. High overlap H3K27me3 peaks cover 242 genes, which amounts to 4% of total gene number **(C)** or 172 repetitive elements, 50% of total repetitive element number **(D)**. Both, loci of hypothetical proteins and transcripts, were included in total gene number.

Cleaning raw sequencing reads, mapping to the reference genome and peak calling with input or H3 sample for normalization, let us identify more than 1300 H3K27me3 peaks per replicate (**Figure 5A**). The peaks cover 14% of total nuclear genome length, with an average peak size of 1792 bp. Good quality of the data was confirmed by a high number of H3K27me3 peaks in the overlaps between the biological replicates, as well as the high correlation values of the reads’ occupancy (Supplementary Figure S3)

Next, we compared H3K27me3-peak coordinates with the loci in *C. merolae* reference genome. We applied a threshold of 50% overlap of the locus length and successfully annotated ca. Six hundred peaks (‘high overlap annotated peaks’) (**Figure 5A**). The remaining peaks corresponded to the reference loci covered by H3K27me3 in less than 50% of their length (‘low overlap annotated peaks’) and to the unannotated peaks without any overlap with reference loci. We concluded that the unannotated peaks are the intergenic regions and/or uncharacterized genomic features missing in the current annotation. Considering the second possibility, we used several strategies to find *de novo* annotation of unknown peaks.

Firstly, we *in silico* translated sequences from the unannotated peaks and searched for open reading frames (ORFs) within them. We found ORFs for all of the unannotated peaks and used them for BLASTP/Pfam searches against the NCBI non-redundant protein sequence database (nr database) or Pfam protein database. Using a specific alignment threshold (alignment length > 50%; *E*-value < 0.5), we could annotate further 178, 164, and 195 peaks for biological replicate 1, 2, and 3, respectively (**Figure 5B**). In the second approach, we extracted DNA sequences of the unknown peaks and used BLASTN searches against the NCBI nucleotide collection (nr/nt database) and NCBI transcript reference sequences (refseq_rna database). The hits surpassing the alignment threshold (alignment length > 50%, *E*-value < 0.5) were compared to BLASTP/Pfam output, which allowed to annotate next 4 to 8 peaks, depending on the biological replicate (**Figure 5B**). In general, our sequence homology searches helped to detect *de novo* annotation of 13–15% peaks from their total number, depending on the biological replicate (**Figure 5A**).

We reasoned that the remaining unknown peaks after *de novo* annotation corresponded to the regions containing promoters and the other regulatory elements of neighboring annotated elements. Given the high density of genes along the chromosomes in *C. merolae* (Matsuzaki et al., 2004), such regions would reside close to 5′ or 3′ gene ends, but would not be present in the annotation. In order to validate this idea, we calculated average distances between gene locations and unannotated peaks. We compared distributions between peaks successfully aligned to BLASTP database (annotated peaks) and the ones showing no significant alignment (unannotated peaks), assuming that BLASTP-aligned peaks truly correspond to novel genes missing in our reference, rather than uncharacterized *cis* elements. However, we did not observe major differences between distances calculated for annotated and unannotated peaks (Supplementary Table S1), suggesting that unannotated peaks do not exclusively correspond to *cis* regulatory elements of known genomic elements. Further development of *C. merolae* genome annotation should deepen our understanding about these remaining uncharacterized H3K27me3 peaks.

### H3K27me3 Genome-wide Distribution – Genomic Feature

Next, we sought to investigate the genomic feature profile underlying H3K27me3 peaks. We picked a stringent group of high overlap annotated targets, removed duplicates (two peaks spanning one genomic element) for each replicate and selected a consensus set of features that appear in any two out of three replicates. As a result, we found that H3K27me3 covers 242 genes, which amounts to 4% of *C. merolae* total gene number (**Figure 5C**). On the other hand, we also detected 172 H3K27me3-bound repetitive elements. Given very low repetitive element occupancy in the *C. merolae* genome (0,7% transposable elements + 5% WSV repeats) (Nozaki et al., 2007), H3K27me3 seems to be predominantly present on repetitive elements, covering as much as 50% of their total number (**Figure 5D**). We noted that the enrichment on both, genes and repetitive elements, is in concordance with the studies in *Drosophila* (Yin et al., 2011). Interestingly, the predominant enrichment of H3K27me3 on repetitive elements was found also in diatom *P. tricornutum* (Veluchamy et al., 2015), suggesting an ancestral role of Polycomb-mediated gene regulation in guarding the genome.

### H3K27me3 Is a Silencing Mark in *C. merolae*

Next, we wanted to characterize the correlation of H3K27me3 enrichment with the expression of its targets. In order to decipher expression level on a genome-wide scale, we performed a RNA-seq experiment on reverse-transcribed RNA extracted from *C. merolae* cultures. The sequencing reads were mapped to the reference genome and expression level was assessed based on the FPKM values. We intersected the FPKM data with H3K27me3-binding profile and distinguished genes and repetitive elements for the analyses.

**FIGURE 6:**
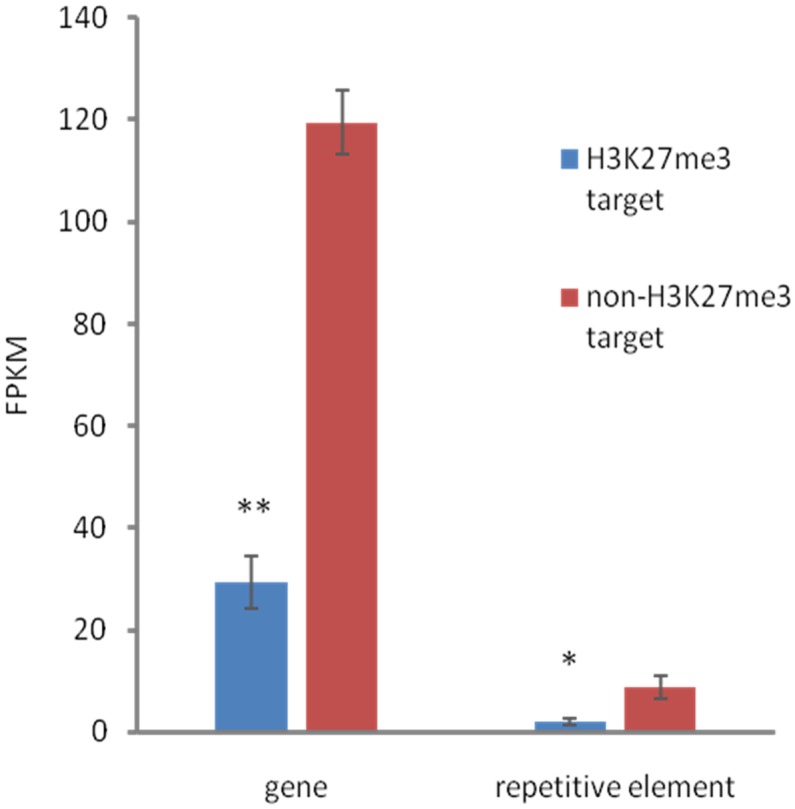
**Relationship between expression and H3K27me3 presence.** H3K27me3 occupancy is anti-correlated with expression level in both, genes and TEs. Expression was estimated based on FPKM values obtained from RNA-seq experiment. Student *t*-test was used to calculate significance. ^∗∗^*p*-value < 0.01, ^∗^*p*-value < 0.05. Error bars represent standard error (SE).

We found that both, H3K27me3-covered genes and H3K27me3-covered repetitive elements are on average significantly less expressed than non-H3K27me3 targets (**Figure 6**), confirming that the H3K27me3 modification in *C. merolae* is highly correlated with gene repression. Moreover, we noted that the general level of repetitive element expression is lower than gene expression, irrespectively of the H3K27me3 status. These results suggest the existence of additional mechanisms involved in repression of the repetitive elements.

### H3K27me3 Gene-body Distribution

**FIGURE 7:**
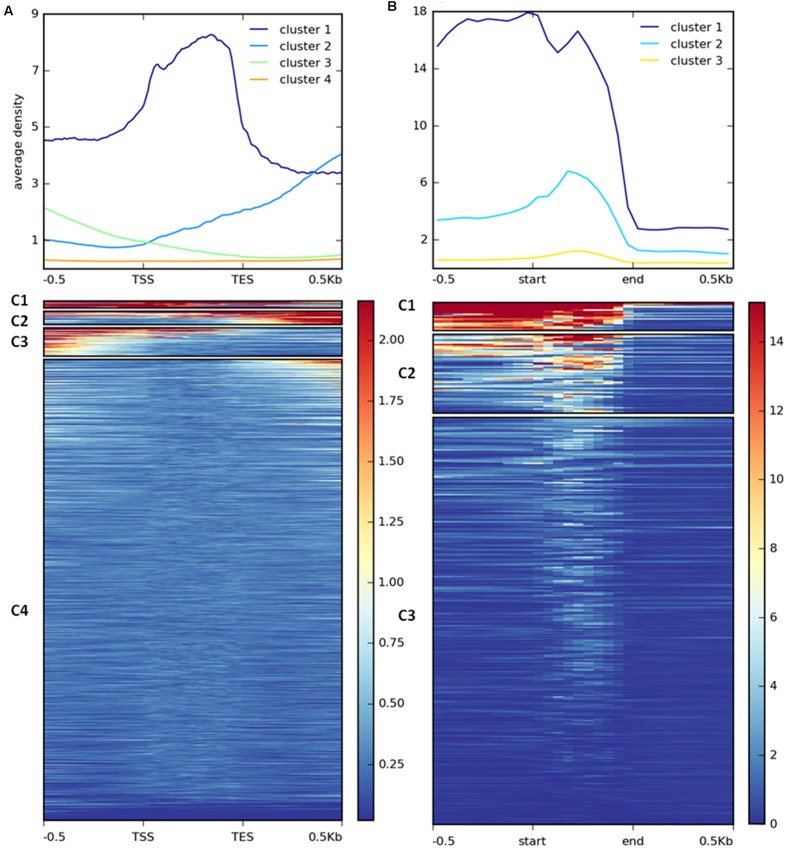
**Clustering of H3K27me3 gene body enrichment.** Coverage of ChIP-seq H3K27me3 reads normalized to input was calculated and used to generate matrix with gene **(A)** or repetitive element **(B)** locations as reference. The reads were scaled to 500 bp windows and flanking regions set to 500 bp. Matrix files served as an input for heatmap generation and K-means clustering in deepTools2.

H3K27me3 enrichment has a broad distribution over gene bodies in plants (Zhang et al., 2007; Yang et al., 2014) or gene bodies and flanking regions in animals (Pauler et al., 2009; Cerase et al., 2014) and diatoms (Veluchamy et al., 2015). To inspect the distribution of the mark in *C. merolae*, we calculated average H3K27me3 enrichment over genic coordinates and observed that H3K27me3 covers gene bodies, 0.5 kb upstream and 0.5 kb downstream regions (Supplementary Figure S2). This general approach obscured whether all targets show similar, broad distribution of H3K27me3 or whether the differential enrichment subgroups are present among the targets. We therefore performed k-means clustering on the results. Our analysis identified four clusters with the preferential presence of H3K27me3 at gene bodies (cluster 1), the 0.5 kb downstream region (cluster 2), and the 0.5 kb upstream region (cluster 3) (**Figure 7A**). Cluster 4 contains non-H3K27me3 targets.

**FIGURE 8:**
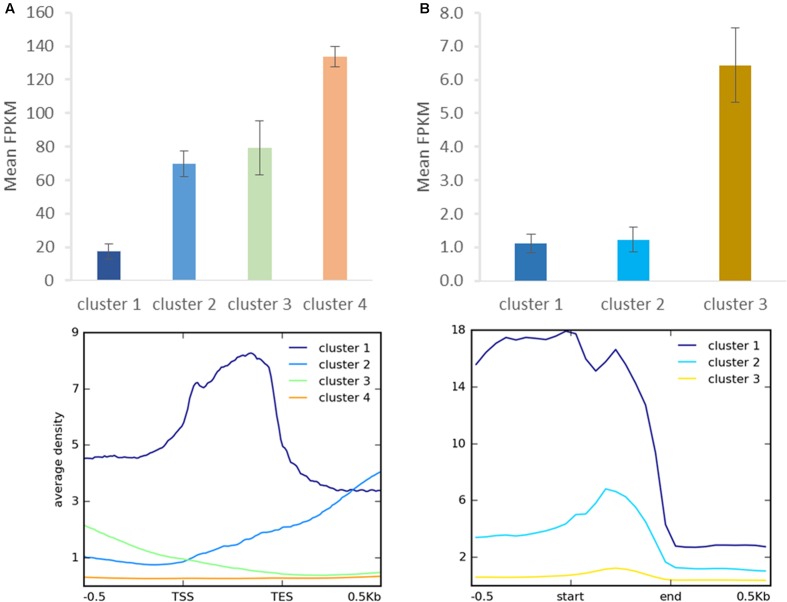
**Relationship between H3K27me3 occupancy and expression after clustering analysis.** Identified clusters (**Figure 7**) were correlated with expression level measured as FPKM in RNA-seq experiment. Expression level was determined for H3K27me3 occupancy clusters from genes **(A)** and repetitive elements **(B)**. Error bars represent standard error.

As distinct H3K27me3 enrichment clusters impact target gene activity differently in some other species (Young et al., 2011; Veluchamy et al., 2015), we asked whether gene expression level varies between the H3K27me3 clusters in *C. merolae*. H3K27me3 differential enrichment compared with RNA-seq data revealed that clusters 1–3 correlate with the gene repression, albeit to an uneven extent. Cluster 1 correlated with the strongest repression level, whereas clusters 2 and 3 correlated with a similar, milder repression (**Figure 8A**). Thus, the strongest silencing effect was seen for H3K27me3 distribution at the gene bodies, similarly to the diatom *P. tricornutum* (Veluchamy et al., 2015), and the H3K27me3 enrichment at the flanking regions is correlated with the milder repression.

We performed similar clustering analysis to characterize H3K27me3 enrichment also on the repetitive elements. We could distinguish two clusters with histone mark presence on 0.5 kb upstream region and gene bodies (cluster 1) or gene bodies only (cluster 2) (**Figure 7B**). Cluster 3 corresponds to non-H3K27me3 targets. In contrast to the genes, we found that cluster 1 and 2 correlate with reduced gene expression to the same extent (**Figure 8B**), suggesting that gene body H3K27me3 distribution has a predominant effect on gene repression. On the other hand, clusters present at repetitive elements are not fully analogous to those at genes as for the former we did not identify a cluster with enrichment exclusively at the flanking regions.

### H3K27me3 Location on Chromosomes

**FIGURE 9:**
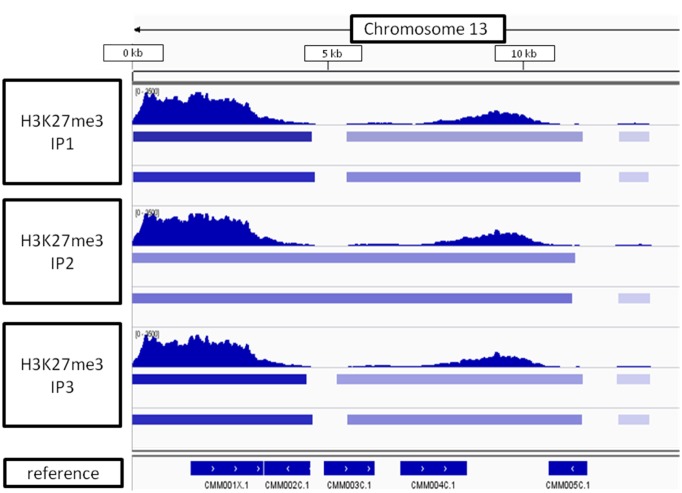
**H3K27me3 domain enrichment on chromosome ends.** 5′ end of chromosome 13 is used as an example. Results from three biological replicates (IP1-3) taken for sequencing are shown. Bars below H3K27me3 tracks correspond to peaks scored by MACS2 with input (top bar) or H3 (bottom bar) used for normalization. The lowest track correspond to *C. merolae* genome annotation (merolae.biol.s.u-tokyo.ac.jp). The data was visualized in IGV v2.3.

Looking at the whole-chromosome level of H3K27me3 distribution, we found a preferential enrichment of the mark on the chromosome ends (**Figure 9**). Such observation concerns 36 out 40 ends from 20 chromosomes. The exceptions, in which the closest H3K27me3 domain was detected only 4–5 kb away from the chromosome end, include: 5′ end of chromosome 3 and 3′ end of chromosomes 7, 14, 16 (Supplementary Table S2 distance). Telomeric repeats in plants span regions from 0.5 kb in *Chlorella vulgaris* to 150 kb in *Nicotiana tabacum* (Fajkus et al., 1995; Higashiyama et al., 1995). *C. merolae* telomere length is relatively short and varies from 400bp to 700bp (Nozaki et al., 2007). Hence, the H3K27me3-domains at the chromosome ends detected in *C. merolae* cover the regions of telomeres and subtelomeres, similarly to what has been shown for the fungi: *N. crassa* (Jamieson et al., 2013) and *Cryptococcus neoformans* (Dumesic et al., 2014).

### H3K27me3-target Gene Ontology

In higher eukaryotes, H3K27me3 is known to target, i.e., developmental, tissue-specific or stress-responsive genes (Boyer et al., 2006; Bracken et al., 2006; Zhang et al., 2007; Lafos et al., 2011; Kleinmanns and Schubert, 2014). We sought to investigate whether similar functional association can be found in *C. merolae*, a unicellular organism with primitive developmental program.

In order to decipher functional classification of H3K27me3 targets, we performed gene ontology analysis with Singular Enrichment Analysis from the AgriGO toolkit (Du et al., 2010). GO classes of H3K27me3-targets and full lists of *C. merolae* genes used as a reference were extracted from the UniProtKB-database. We found a GO annotation for 3085 genes (50,5% from total number of 6108, including hypothetical proteins and transcripts) in the reference with 144 GO-annotated genes among H3K27me3-targets (62,6% from total number of 230). Statistical significance was determined based on the adjusted *p*-value with Yekutieli test for FDR. For statistical tests, we selected only those GO classes that were represented by at least five entries (see material and methods). All three different GO sub-ontologies were taken into account: molecular function (GO:0003674), biological process (GO::0008150), and cellular component (GO::0008372).

**FIGURE 10:**
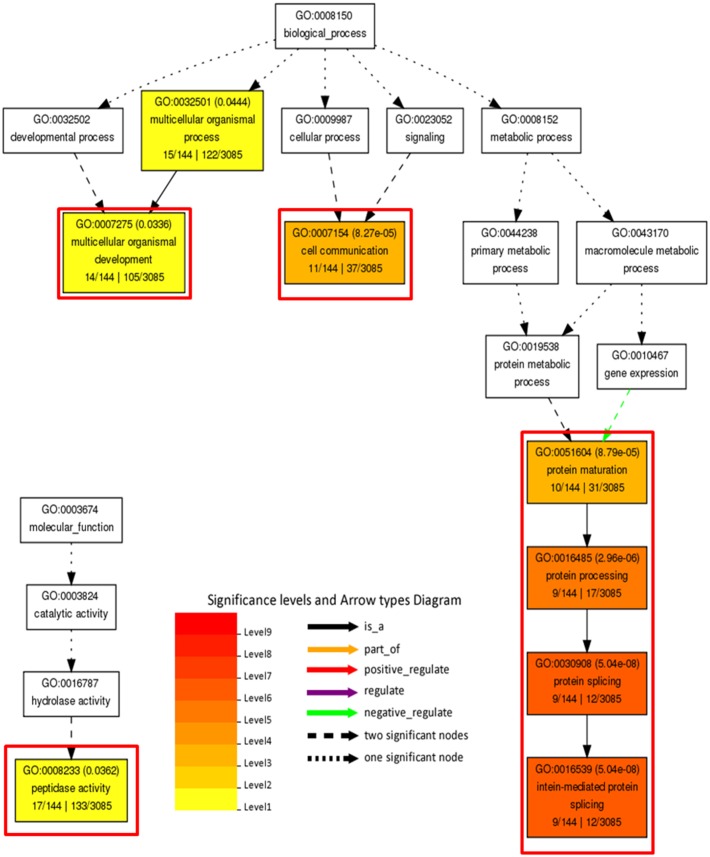
**Gene ontology analysis on H3K27me3 target genes.** Significantly enriched terms from molecular function and biological process subontologies (no significant hits found for cellular component subontology). Color coding represents significance level (1–9) below adjusted *p*-value threshold (*p* < 0.05). Adjusted *p*-values are shown next to GO IDs for each GO term. Relationships between GO terms are reflected in the arrow types. Red frames highlight GO terms connected with intein-mediated protein splicing. Numbers below subcategory name correspond to: GO subcategory gene number in query/total gene number in query | GO subcategory gene number in reference (TAIR10)/total gene number in reference.

We did not reveal any significantly (adjusted *p*-value < 0.05) enriched GO terms in cellular component sub-ontology. In contrast, we detected three GO term branches in biological process and one in molecular function sub-ontologies (**Figure 10**). Consistently with the known H3K27me3 function in the repression of developmental programs, we detected significantly enriched GO terms related to organismal process and development. Surprisingly, among enriched terms we found also those corresponding to protein maturation and intein-mediated protein splicing.

Protein splicing is a protein maturation mechanism based on the excision of a protein fragment (intein) from a precursor and ligation of the flanking polypeptides (exteins) (Topilina and Mills, 2014). Comparative analyses of amino acid sequences revealed a homology of protein splice-junction motif from inteins to the C-terminal Hog domains in Hedgehog proteins, the secretory proteins controlling developmental processes in Metazoa (Jiang and Hui, 2008).

In concordance to the protein splicing mechanism and the function of intein-containing genes, we observed that the majority of protein entries present in significantly enriched GO terms (organismal process and development, cell communication and peptidase function) overlap with the protein splicing group (Supplementary Table S3). Moreover, we found that all of the H3K27me3 targets present in the protein splicing term indeed possess a Hog domain as the Hedgehog proteins.

Interestingly, the genes encoding Hedgehog proteins in *C. merolae* reside in the telomeric and subtelomeric regions of the chromosomes (Nozaki et al., 2007), which is in agreement to our results on the preferential H3K27me3 enrichment at the chromosome ends (**Figure 9**). Moreover, comparing with clustering data, we observed that Hedgehog loci were enriched in cluster 3 (Supplementary Table S4), bound by H3K27me3 preferentially to the upstream regions and moderately repressed.

## Discussion

Polycomb-group mediated regulation of gene expression in multicellular organisms is intensively studied in plants and has important roles in stress responses and developmental phase transitions (Köhler and Villar, 2008; Kleinmanns and Schubert, 2014). However, a model for Pc-G function in unicellular, photosynthetic organisms is currently lacking. We therefore performed homology searches and Bayesian phylogenetic tree analyses on the conserved alignment blocks. Our results confirmed a frequent absence of the PRC1 complex, ancient origin of PRC2 complex and the widespread distribution of core components of PRC2 in the green lineage [see also: (Shaver et al., 2010; Kim et al., 2013)]. We detected the PcG mark H3K27me3 in prasinophytes and red algae, arguing for the functionality of PRC2 in these organisms. Subsequent analyses of H3K27me3 occupancy in the model red alga, *C. merolae*, and correlative analyses with transcriptomic data revealed several important observations in this unicellular organism.

We observed that H3K27me3 in *C. merolae* is present on both, genes and repetitive elements, covering 4% of total gene number and 50% of total repetitive element number. The occupancy on both genomic types is in the agreement with the results from *Drosophila* and *Phaeodactylum*, suggesting an ancestral function of PcG in repetitive element silencing. As *Arabidopsis* H3K27me3 occupies preferentially genes, our results suggest also a partial divergence from PcG-mediated repetitive element repression in higher plants.

In *Arabidopsis* endosperm, the vicinity of repetitive elements was shown to impact H3K27me3-mediated gene imprinting (Weinhofer et al., 2010; Wolff et al., 2011). In contrast, the H3K27me3-covered genes in *C. merolae* are not in the vicinity of repetitive elements. However, we cannot exclude the possibility that the genome annotation is incomplete, with unknown types of repetitive elements being not reported.

We showed that H3K27me3 occupancy anti-correlates with the expression of its targets, consistently with its function in the multicellular organisms. As the other lysine-27 methylation marks, H3K27me1 and H3K27me2, have a repressive function in higher plants (Bastow et al., 2004; Jacob et al., 2009), similarly to the H3K27me3, the future steps should include profiling of those modifications in *C. merolae* genome. We also observed that the degree of repression is tightly correlated with the H3K27me3 profile at the genic level. The lowest expression was found for the genes covered by H3K27me3 over the gene body, whereas the targets with H3K27me3 occupancy on 5′ and 3′ flanking regions associate with intermediate repression.

Furthermore, H3K27me3 shows an enrichment on chromosome ends and covers a broad region including telomeric repeats and subtelomeric genes in *C. merolae*. Interestingly, several other species were reported to show an enrichment of H3K27me3 at the subtelomeric and telomeric regions (Smith et al., 2008; Vaquero-sedas et al., 2012). Moreover, Polycomb proteins were proved to be associated with telomere-binding factors (Zhou et al., 2013) and telobox motifs demonstrated to be enriched at PcG-peaks (Deng et al., 2013), arguing for a functional connection between PcG-mediated repression and chromosomal ends.

Our gene ontology analysis showed that H3K27me3 covers genes associated with intein-mediated protein splicing. Inteins are considered as ancient mobile elements present in all three domains of life (eukaryotes, archaea, bacteria) and viruses (Pietrokovski, 2001; Novikova et al., 2014). Hence, our results suggest that the H3K27me3-mediated repression might function in guarding the *C. merolae* genome. Such notion would be consistent with the predominant enrichment of H3K27me3 on the repetitive elements (**Figure 5D**). On the other hand, inteins may also develop to become post-translational regulatory elements in the course of evolution, as seen for the conditional protein splicing of Hog domains in animals (Shah and Muir, 2014). Therefore, it is of special importance to decipher the function of intein-containing Hedgehog genes and the conditions for their de-repression in *C. merolae.*

Interestingly, we also observed that the intein-containing genes reside in the subtelomeric regions and are moderately repressed by H3K27me3 that occupies upstream regions from their TSS. It is currently unclear how protein splicing, moderate H3K27me3-mediated repression and subtelomeric localization are inter-connected. It is feasible that the integration of intein-containing genes appeared relatively recently and that these ‘young’ H3K27me3 targets display H3K27me3 enrichment at regions upstream of their TSS, in agreement with their moderate repression. Comparative genomics between *C. merolae* and related species should be used to validate this idea. In turn, subtelomeres are dynamic regions and the objects of ectopic recombination events, which facilitate gene diversification, phenotypic diversity and adaptation to different environments, exemplified by an olfactory receptor gene family in humans (Linardopoulou et al., 2001), contingency systems in various pathogens (Barry et al., 2003) and carbon-source metabolism genes in *S. cerevisiae* (Carlson and Botstein, 1983; Louis et al., 1994; Brown et al., 2010). Hence, it is possible that the intein-containing subtelomeric H3K27me3-covered genes are the targets of rapid adaptive evolution. Characterization of their duplication frequency and diversification within their gene families should validate such notion.

Last but not the least, our results on H3K27me3 presence in various algae and genome-wide characterization of expression status and H3K27me3 occupancy in the model red alga, *C. merolae*, provide a resource for chromatin and transcriptomic studies. An existence of genetic tools, optimized growth conditions and the sequenced genome in *C. merolae* offer a possibility to decipher genome regulation in a broad evolutionary context, including the organisms with super-reduced genomes.

## Author Contributions

PM, OK, and DS designed the research. FF and AW designed *C. merolae* growth scheme. FF handled *C. merolae* cell cultures. PM performed bioinformatic analyses, chromatin immunoprecipitations and sequencing library preparations. OK performed competition assay and western blot experiments. PM and DS performed homology searches for H3K27me3 targets used in ChIP-RT-qPCR. The manuscript was written by PM and revised by the other authors.

## Conflict of Interest Statement

The authors declare that the research was conducted in the absence of any commercial or financial relationships that could be construed as a potential conflict of interest.

## Footnotes

1 czon.jp/download/annot_list.txt

## References

[B1] Abascal F. Zardoya R. Posada D. Miller C. J. Lou H. J. Johnson A. D. (2005). ProtTest: selection of best-fit models of protein evolution. *Bioinformatics* 21 2104–2105. 10.1093/bioinformatics/bti263 15647292

[B2] Barry J. D. Ginger M. L. Burton P. McCulloch R. (2003). Why are parasite contingency genes often associated with telomeres? *Int. J. Parasitol.* 33 29–45. 10.1016/S0020-7519(02)00247-3 12547344

[B3] Bastow R. Mylne J. S. Lister C. Lippman Z. Martienssen R. A. Dean C. (2004). Vernalization requires epigenetic silencing of FLC by histone methylation. *Nature* 427 164–167. 10.1038/nature02269 14712277

[B4] Berke L. Snel B. (2015). The plant Polycomb repressive complex 1 (PRC1) existed in the ancestor of seed plants and has a complex duplication history. *BMC Evol. Biol.* 15:44 10.1186/s12862-015-0319-z PMC4397884 25881027

[B5] Bowman J. L. Floyd S. K. Sakakibara K. (2007). Green genes-comparative genomics of the green branch of life. *Cell* 129 229–234. 10.1016/j.cell.2007.04.004 17448980

[B6] Boyer L. A. Plath K. Zeitlinger J. Brambrink T. Medeiros L. A. Lee T. I. (2006). Polycomb complexes repress developmental regulators in murine embryonic stem cells. *Nature* 441 349–353. 10.1038/nature04733 16625203

[B7] Bracken A. P. Dietrich N. Pasini D. Hansen K. H. Helin K. (2006). Genome-wide mapping of Polycomb target genes unravels their roles in cell fate transitions. *Genes Dev.* 20 1123–1136. 10.1101/gad.381706.present 16618801 PMC1472472

[B8] Brown C. A. Murray A. W. Verstrepen K. J. (2010). Rapid expansion and functional divergence of subtelomeric gene families in yeasts. *Curr. Biol.* 20 895–903. 10.1016/j.cub.2010.04.027 20471265 PMC2877759

[B9] Butenko Y. Ohad N. (2011). Polycomb-group mediated epigenetic mechanisms through plant evolution. *Biochim. Biophys. Acta* 1809 395–406. 10.1016/j.bbagrm.2011.05.013 21664995

[B10] Camacho C. Coulouris G. Avagyan V. Ma N. Papadopoulos J. Bealer K. (2009). BLAST+: architecture and applications. *BMC Bioinformatics* 10:421 10.1186/1471-2105-10-421 PMC2803857 20003500

[B11] Carlson M. Botstein D. (1983). Organization of the *SUC* gene family in *Saccharomyces*. *Mol. Cell. Biol.* 3 351–359. 10.1128/MCB.3.3.351 6843548 PMC368543

[B12] Cerase A. Smeets D. Tang Y. A. Gdula M. Kraus F. Spivakov M. (2014). Spatial separation of Xist RNA and polycomb proteins revealed by superresolution microscopy. *Proc. Natl. Acad. Sci. U.S.A.* 111 2235–2240. 10.1073/pnas.1312951111 24469834 PMC3926064

[B13] Chen D. Huang Y. Ruan Y. Shen W. H. (2016). The evolutionary landscape of PRC1 core components in green lineage. *Planta* 243 825–846. 10.1007/s00425-015-2451-9 26729480

[B14] Cui J. Zhang Z. Shao Y. Zhang K. Leng P. Liang Z. (2015). Genome-wide identification, evolutionary, and expression analyses of histone H3 variants in plants. *Biomed. Res. Int.* 2015:341598 10.1155/2015/341598 PMC4357034 25815311

[B15] Del Prete S. Mikulski P. Schubert D. Gaudin V. (2015). One, two, three: Polycomb proteins hit all dimensions of gene regulation. *Genes* 6 520–542. 10.3390/genes6030520 26184319 PMC4584315

[B16] Deleris A. Stroud H. Bernatavichute Y. Johnson E. Klein G. Schubert D. (2012). Loss of the DNA methyltransferase MET1 Induces H3K9 hypermethylation at PcG target genes and redistribution of H3K27 trimethylation to transposons in *Arabidopsis thaliana*. *PLoS Genet.* 8:e1003062 10.1371/journal.pgen.1003062 PMC3510029 23209430

[B17] Deng W. Buzas D. M. Ying H. Robertson M. Taylor J. Peacock W. J. (2013). Arabidopsis Polycomb Repressive Complex 2 binding sites contain putative GAGA factor binding motifs within coding regions of genes. *BMC Genomics* 14:593 10.1186/1471-2164-14-593 PMC3766684 24001316

[B18] Derkacheva M. Hennig L. (2014). Variations on a theme: Polycomb group proteins in plants. *J. Exp. Bot.* 65 2769–2784. 10.1093/jxb/ert410 24336446

[B19] Du Z. Zhou X. Ling Y. Zhang Z. Su Z. (2010). agriGO: a GO analysis toolkit for the agricultural community. *Nucleic Acids Res.* 38 W64–W70. 10.1093/nar/gkq310 20435677 PMC2896167

[B20] Dumesic P. A. Homer C. M. Moresco J. J. Pack L. R. Shanle E. K. Coyle S. M. (2014). Product binding enforces the genomic specificity of a yeast polycomb repressive complex. *Cell* 160 204–218. 10.1016/j.cell.2014.11.039 25533783 PMC4303595

[B21] Ebert A. Schotta G. Lein S. Kubicek S. Krauss V. Jenuwein T. (2004). Su(var) genes regulate the balance between euchromatin and heterochromatin in *Drosophila*. *Genes Dev.* 18 2973–2983. 10.1101/gad.323004 15574598 PMC534657

[B22] Fajkus J. Kovarík A. Královics R. Bezděk M. (1995). Organization of telomeric and subtelomeric chromatin in the higher plant *Nicotiana tabacum*. *Mol. Gen. Genet.* 247 633–638. 10.1007/BF00290355 7603443

[B23] Ferrari K. J. Scelfo A. Jammula S. Cuomo A. Barozzi I. Stützer A. (2014). Polycomb-dependent H3K27me1 and H3K27me2 regulate active transcription and enhancer fidelity. *Mol. Cell* 53 49–62. 10.1016/j.molcel.2013.10.030 24289921

[B24] Hennig L. Derkacheva M. (2009). Diversity of Polycomb group complexes in plants: same rules, different players? *Trends Genet.* 25 414–423. 10.1016/j.tig.2009.07.002 19716619

[B25] Higashiyama T. Maki S. Yamada T. (1995). Molecular organization of *Chlorella vulgaris* chromosome I: presence of telomeric repeats that are conserved in higher plants. *Mol. Gen. Genet.* 246 29–36. 10.1007/BF00290130 7823910

[B26] Huang Y. Chen D.-H. Liu B.-Y. Shen W.-H. Ruan Y. (2016). Conservation and diversification of polycomb repressive complex 2 (PRC2) proteins in the green lineage. *Brief. Funct. Genomics* 16 106–119. 10.1093/bfgp/elw007 27032420

[B27] Jacob Y. Feng S. LeBlanc C. A. Bernatavichute Y. V. Stroud H. Cokus S. (2009). ATXR5 and ATXR6 are H3K27 monomethyltransferases required for chromatin structure and gene silencing. *Nat. Struct. Mol. Biol.* 16 763–768. 10.1038/nsmb.1611 19503079 PMC2754316

[B28] Jacob Y. Michaels S. D. (2009). H3K27me1 is E(z) in animals, but not in plants. *Epigenetics* 4 366–369. 10.4161/epi.4.6.9713 19736521 PMC2814773

[B29] Jamieson K. Rountree M. R. Lewis Z. A. Stajich J. E. Selker E. U. (2013). Regional control of histone H3 lysine 27 methylation in *Neurospora*. *Proc. Natl. Acad. Sci. U.S.A.* 110 6027–6032. 10.1073/pnas.1303750110 23530226 PMC3625340

[B30] Jeffery C. J. (2003). Moonlighting proteins: old proteins learning new tricks. *Trends Genet.* 19 415–417. 10.1016/S0168-9525(03)00167-7 12902157

[B31] Jiang J. Hui C.-C. (2008). Hedgehog signaling in development and cancer. *Dev. Cell* 15 801–812. 10.1016/j.devcel.2008.11.010 19081070 PMC6443374

[B32] Kim E. Ma X. Cerutti H. (2013). Gene silencing in microalgae?: mechanisms and biological roles. *Bioresour. Technol.* 184 23–32. 10.1016/j.biortech.2014.10.119 25466994

[B33] Kleinmanns J. A. Schubert D. (2014). Polycomb and Trithorax group protein-mediated control of stress responses in plants. *Biol. Chem.* 395 1291–1300. 10.1515/hsz-2014-0197 25153238

[B34] Köhler C. Villar C. B. R. (2008). Programming of gene expression by Polycomb group proteins. *Trends Cell Biol.* 18 236–243. 10.1016/j.tcb.2008.02.005 18375123

[B35] Lachner M. Sengupta R. Schotta G. Jenuwein T. (2004). Trilogies of histone lysine methylation as epigenetic landmarks of the eukaryotic genome. *Cold Spring Harb. Symp. Quant. Biol.* 69 209–218. 10.1101/sqb.2004.69.209 16117651

[B36] Lafos M. Kroll P. Hohenstatt M. L. Thorpe F. L. Clarenz O. Schubert D. (2011). Dynamic regulation of H3K27 trimethylation during *Arabidopsis* differentiation. *PLoS Genet.* 7:e1002040 10.1371/journal.pgen.1002040 PMC3072373 21490956

[B37] Larkin M. A. Blackshields G. Brown N. P. Chenna R. Mcgettigan P. A. McWilliam H. (2007). Clustal W and Clustal X version 2.0. *Bioinformatics* 23 2947–2948. 10.1093/bioinformatics/btm404 17846036

[B38] Linardopoulou E. Mefford H. C. Nguyen O. Friedman C. van den Engh G. Farwell D. G. (2001). Transcriptional activity of multiple copies of a subtelomerically located olfactory receptor gene that is polymorphic in number and location. *Hum. Mol. Genet.* 10 2373–2383. 10.1093/hmg/10.21.2373 11689484

[B39] Lindroth A. M. Shultis D. Jasencakova Z. Fuchs J. Johnson L. Schubert D. (2004). Dual histone H3 methylation marks at lysines 9 and 27 required for interaction with CHROMOMETHYLASE3. *EMBO J.* 23 4286–4296. 10.1038/sj.emboj.7600430 15457214 PMC524394

[B40] Liu Y. Taverna S. D. Muratore T. L. Shabanowitz J. Hunt D. F. Allis C. D. (2007). RNAi-dependent H3K27 methylation is required for heterochromatin formation and DNA elimination in *Tetrahymena*. *Genes Dev.* 21 1530–1545. 10.1101/gad.1544207 17575054 PMC1891430

[B41] Louis E. J. Naumova E. S. Lee A. Naumov G. Haber J. E. (1994). The chromosome end in yeast: its mosaic nature and influence on recombinational dynamics. *Genetics* 136 789–802. 8005434 10.1093/genetics/136.3.789 PMC1205885

[B42] Matsuzaki M. Misumi O. Shin-I T. Maruyama S. Takahara M. Miyagishima S.-Y. (2004). Genome sequence of the ultrasmall unicellular red alga *Cyanidioschyzon merolae* 10D. *Nature* 428 653–657. 10.1038/nature02398 15071595

[B43] McKay D. J. Klusza S. Penke T. J. R. Meers M. P. Curry K. P. McDaniel S. L. (2015). Interrogating the function of metazoan histones using engineered gene clusters. *Dev. Cell* 32 373–386. 10.1016/j.devcel.2014.12.025 25669886 PMC4385256

[B44] Merchant S. S. Prochnik S. E. Vallon O. Harris E. H. Karpowicz J. Witman G. B. (2010). The *Chlamydomonas* genome reveals the evolution of key animal and plant functions. *Science* 318 245–250. 10.1126/science.1143609.The PMC2875087 17932292

[B45] Müller J. Hart C. M. Francis N. J. Vargas M. L. Sengupta A. Wild B. (2002). Histone methyltransferase activity of a *Drosophila* Polycomb group repressor complex. *Cell* 111 197–208. 10.1016/S0092-8674(02)00976-5 12408864

[B46] Müller J. Verrijzer P. (2009). Biochemical mechanisms of gene regulation by polycomb group protein complexes. *Curr. Opin. Genet. Dev.* 19 150–158. 10.1016/j.gde.2009.03.001 19345089

[B47] Nekrasov M. Klymenko T. Fraterman S. Papp B. Oktaba K. Köcher T. (2007). Pcl-PRC2 is needed to generate high levels of H3-K27 trimethylation at Polycomb target genes. *EMBO J.* 26 4078–4088. 10.1038/sj.emboj.7601837 17762866 PMC1964751

[B48] Novikova O. Topilina N. Belfort M. (2014). Enigmatic distribution, evolution, and function of inteins. *J. Biol. Chem.* 289 14490–14497. 10.1074/jbc.R114.548255 24695741 PMC4031506

[B49] Nozaki H. Takano H. Misumi O. Terasawa K. Matsuzaki M. Maruyama S. (2007). A 100%-complete sequence reveals unusually simple genomic features in the hot-spring red alga *Cyanidioschyzon merolae*. *BMC Biol.* 5:28 10.1186/1741-7007-5-28 PMC1955436 17623057

[B50] Okada T. Endo M. Singh M. B. Bhalla P. L. (2005). Analysis of the histone H3 gene family in Arabidopsis and identification of the male-gamete-specific variant *AtMGH3*. *Plant J.* 44 557–568. 10.1111/j.1365-313X.2005.02554.x 16262706

[B51] Palenik B. Grimwood J. Aerts A. Rouzé P. Salamov A. Putnam N. (2007). The tiny eukaryote *Ostreococcus* provides genomic insights into the paradox of plankton speciation. *Proc. Natl. Acad. Sci. U.S.A.* 104 7705–7710. 10.1073/pnas.0611046104 17460045 PMC1863510

[B52] Papp B. Müller J. (2006). Histone trimethylation and the maintenance of transcriptional ON and OFF states by trxG and PcG proteins. *Genes Dev.* 20 2041–2054. 10.1101/gad.388706 16882982 PMC1536056

[B53] Park S. Oh S. van Nocker S. (2012). Genomic and gene-level distribution of histone H3 dimethyl lysine-27 (H3K27me2) in Arabidopsis. *PLoS ONE* 7:e52855 10.1371/journal.pone.0052855 PMC3532402 23285203

[B54] Pauler F. M. Sloane M. A. Huang R. Regha K. Koerner M. V. Tamir I. (2009). H3K27me3 forms BLOCs over silent genes and intergenic regions and specifies a histone banding pattern on a mouse autosomal chromosome. *Genome Res.* 19 221–233. 10.1101/gr.080861.108.4 19047520 PMC2652204

[B55] Pietrokovski S. (2001). Intein spread and extinction in evolution. *Trends Genet.* 17 465–472. 10.1016/S0168-9525(01)02365-4 11485819

[B56] Price D. C. Chan C. X. Yoon H. S. Yang E. C. Qiu H. Weber A. P. (2012). Cyanophora paradoxa genome elucidates origin of photosynthesis in algae and plants. *Science* 335 843–847. 10.1126/science.1213561 22344442

[B57] Quinlan A. R. Hall I. M. (2010). BEDTools: a flexible suite of utilities for comparing genomic features. *Bioinformatics* 26 841–842. 10.1093/bioinformatics/btq033 20110278 PMC2832824

[B58] Rambaut A. (2009). *FigTree, a Graphical Viewer of Phylogenetic Trees*. Available at: http://tree.bio.ed.ac.uk/software/figtree/

[B59] Ramirez F. Ryan D. P. Gruning B. Bhardwaj V. Kilpert F. Richter A. S. (2016). deepTools2: a next generation web server for deep-sequencing data analysis. *Nucleic Acids Res.* 44 160–165. 10.1093/nar/gkw257 PMC4987876 27079975

[B60] Ronquist F. Teslenko M. Van Der Mark P. Ayres D. L. Darling A. Höhna S. (2012). Mrbayes 3.2: efficient Bayesian phylogenetic inference and model choice across a large model space. *Syst. Biol.* 61 539–542. 10.1093/sysbio/sys029 22357727 PMC3329765

[B61] Schuettengruber B. Chourrout D. Vervoort M. Leblanc B. Cavalli G. (2007). Genome regulation by Polycomb and Trithorax proteins. *Cell* 128 735–745. 10.1016/j.cell.2007.02.009 17320510

[B62] Schwartz Y. B. Pirrotta V. (2008). Polycomb complexes and epigenetic states. *Curr. Opin. Cell Biol.* 20 266–273. 10.1016/j.ceb.2008.03.002 18439810

[B63] Schwartz Y. B. Pirrotta V. (2013). A new world of Polycombs: unexpected partnerships and emerging functions. *Nat. Rev. Genet.* 14 853–864. 10.1038/nrg3603 24217316

[B64] Shah N. H. Muir T. W. (2014). Inteins: nature’s gift to protein chemists. *Chem. Sci.* 5 446–461. 10.1039/C3sc52951g 24634716 PMC3949740

[B65] Shaver S. Casas-Mollano J. A. Cerny R. L. Cerutti H. (2010). Origin of the polycomb repressive complex 2 and gene silencing by an E(z) homolog in the unicellular alga *Chlamydomonas*. *Epigenetics* 5 301–312. 10.4161/epi.5.4.11608 20421736

[B66] Smith K. M. Kothe G. O. Matsen C. B. Khlafallah T. K. Adhvaryu K. K. Hemphill M. (2008). The fungus *Neurospora crassa* displays telomeric silencing mediated by multiple sirtuins and by methylation of histone H3 lysine 9. *Epigenetics Chromatin* 1:5 10.1186/1756-8935-1-5 PMC2596135 19014414

[B67] Sung S. Schmitz R. J. Amasino R. M. (2006). A PHD finger protein involved in both the vernalization and photoperiod pathways in *Arabidopsis*. *Genes Dev.* 20 3244–3248. 10.1101/gad.1493306 17114575 PMC1686601

[B68] Talavera G. Castresana J. (2007). Improvement of phylogenies after removing divergent and ambiguously aligned blocks from protein sequence alignments. *Syst. Biol.* 56 564–577. 10.1080/10635150701472164 17654362

[B69] Thorvaldsdóttir H. Robinson J. T. Mesirov J. P. (2013). Integrative Genomics Viewer (IGV): high-performance genomics data visualization and exploration. *Brief. Bioinform.* 14 178–192. 10.1093/bib/bbs017 22517427 PMC3603213

[B70] Topilina N. I. Mills K. V. (2014). Recent advances in *in vivo* applications of intein-mediated protein splicing. *Mob. DNA* 5:5 10.1186/1759-8753-5-5 PMC3922620 24490831

[B71] Trapnell C. Roberts A. Goff L. Pertea G. Kim D. Kelley D. R. (2012). Differential gene and transcript expression analysis of RNA-seq experiments with TopHat and Cufflinks. *Nat. Protoc.* 7 562–578. 10.1038/nprot.2012.016 22383036 PMC3334321

[B72] Vaquero-sedas I. Luo C. Vega-palas M. A. (2012). Analysis of the epigenetic status of telomeres by using ChIP-seq data. *Nucleic Acids Res.* 40:e163 10.1093/nar/gks730 PMC3505975 22855559

[B73] Veerappan C. S. Avramova Z. Moriyama E. N. (2008). Evolution of SET-domain protein families in the unicellular and multicellular Ascomycota fungi. *BMC Evol. Biol.* 8:190 10.1186/1471-2148-8-190 PMC2474616 18593478

[B74] Veluchamy A. Rastogi A. Lin X. Lombard B. Murik O. Thomas Y. (2015). An integrative analysis of post-translational histone modifications in the marine diatom *Phaeodactylum tricornutum*. *Genome Biol.* 16:102 10.1186/s13059-015-0671-8 PMC4504042 25990474

[B75] Waterhouse A. M. Procter J. B. Martin D. M. A. Clamp M. Barton G. J. (2009). Jalview Version 2–A multiple sequence alignment editor and analysis workbench. *Bioinformatics* 25 1189–1191. 10.1093/bioinformatics/btp033 19151095 PMC2672624

[B76] Weinhofer I. Hehenberger E. Roszak P. Hennig L. Köhler C. (2010). H3K27me3 profiling of the endosperm implies exclusion of polycomb group protein targeting by DNA methylation. *PLoS Genet.* 6:e1001152 10.1371/journal.pgen.1001152 PMC2951372 20949070

[B77] Wolff P. Weinhofer I. Seguin J. Roszak P. Beisel C. Donoghue M. T. A. (2011). High-resolution analysis of parent-of-origin allelic expression in the Arabidopsis endosperm. *PLoS Genet.* 7:e1002126 10.1371/journal.pgen.1002126 PMC3116908 21698132

[B78] Worden A. Z. Lee J.-H. Mock T. Rouzé P. Simmons M. P. Aerts A. L. (2009). Green evolution and dynamic adaptations revealed by genomes of the marine picoeukaryotes *Micromonas*. *Science* 324 268–272. 10.1126/science.1167222 19359590

[B79] Yang C. Bratzel F. Hohmann N. Koch M. Turck F. Calonje M. (2013). VAL-and AtBMI1-mediated H2Aub initiate the switch from embryonic to postgerminative growth in *Arabidopsis*. *Curr. Biol.* 23 1324–1329. 10.1016/j.cub.2013.05.050 23810531

[B80] Yang H. Howard M. Dean C. (2014). Antagonistic roles for H3K36me3 and H3K27me3 in the cold-induced epigenetic switch at *Arabidopsis* FLC. *Curr. Biol.* 24 1793–1797. 10.1016/j.cub.2014.06.047 25065750 PMC4123163

[B81] Yin H. Sweeney S. Raha D. Snyder M. Lin H. (2011). A high-resolution whole-genome map of key chromatin modifications in the adult *Drosophila melanogaster*. *PLoS Genet.* 7:e1002380 10.1371/journal.pgen.1002380 PMC3240582 22194694

[B82] Young M. D. Willson T. A. Wakefield M. J. Trounson E. Hilton D. J. Blewitt M. E. (2011). ChIP-seq analysis reveals distinct H3K27me3 profiles that correlate with transcriptional activity. *Nucleic Acids Res.* 39 7415–7427. 10.1093/nar/gkr416 21652639 PMC3177187

[B83] Zhang X. Clarenz O. Cokus S. Bernatavichute Y. V. Pellegrini M. Goodrich J. (2007). Whole-genome analysis of histone H3 lysine 27 trimethylation in *Arabidopsis*. *PLoS Biol.* 5:e129 10.1371/journal.pbio.0050129 PMC1852588 17439305

[B84] Zhou Y. Hartwig B. James G. V. Schneeberger K. Turck F. (2013). Complementary activities of TELOMERE REPEAT BINDING proteins and polycomb group complexes in transcriptional regulation of target genes. *Plant Cell.* 28 87–101. 10.1105/tpc.15.00787 PMC4746681 26721861

