# Supplementary material for: Characterization of the Polycomb-Group Mark H3K27me3 in Unicellular Algae
Source: Front Plant Sci. 2017 Apr 26;8:607. doi: 10.3389/fpls.2017.00607 (PMC5405695; doi:10.3389/fpls.2017.00607)
Supplement: Supplementary file 3 [file Data_Sheet_1.pdf]

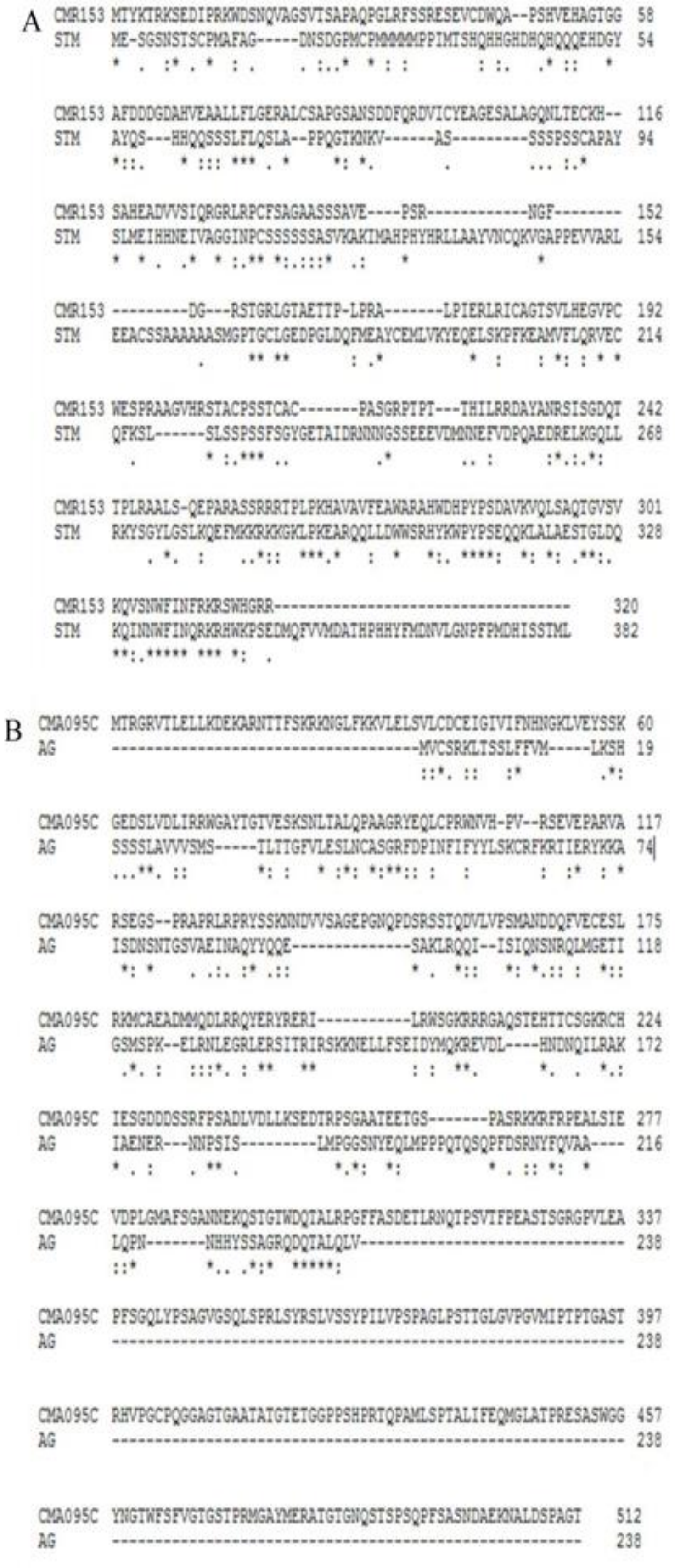

Figure S1. Alignment of selected H3K27me3 targets from *A. thaliana*: (A) SHOOT MERISTEMLESS (STM) and (B) AGAMOUS (AG), and their homologs from *C. merolae*.

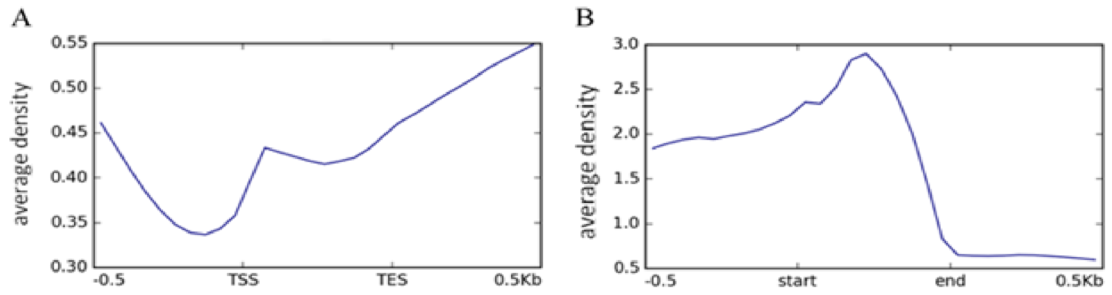

Figure S2. Average H3K27me3 occupancy over genes (A) and repetitive elements (B). The H3K27me3 reads were scaled to 500bp windows and flanking regions set to 0.5kb. DeepTools2 package was used to compute an abundance of the reads and plot creation.

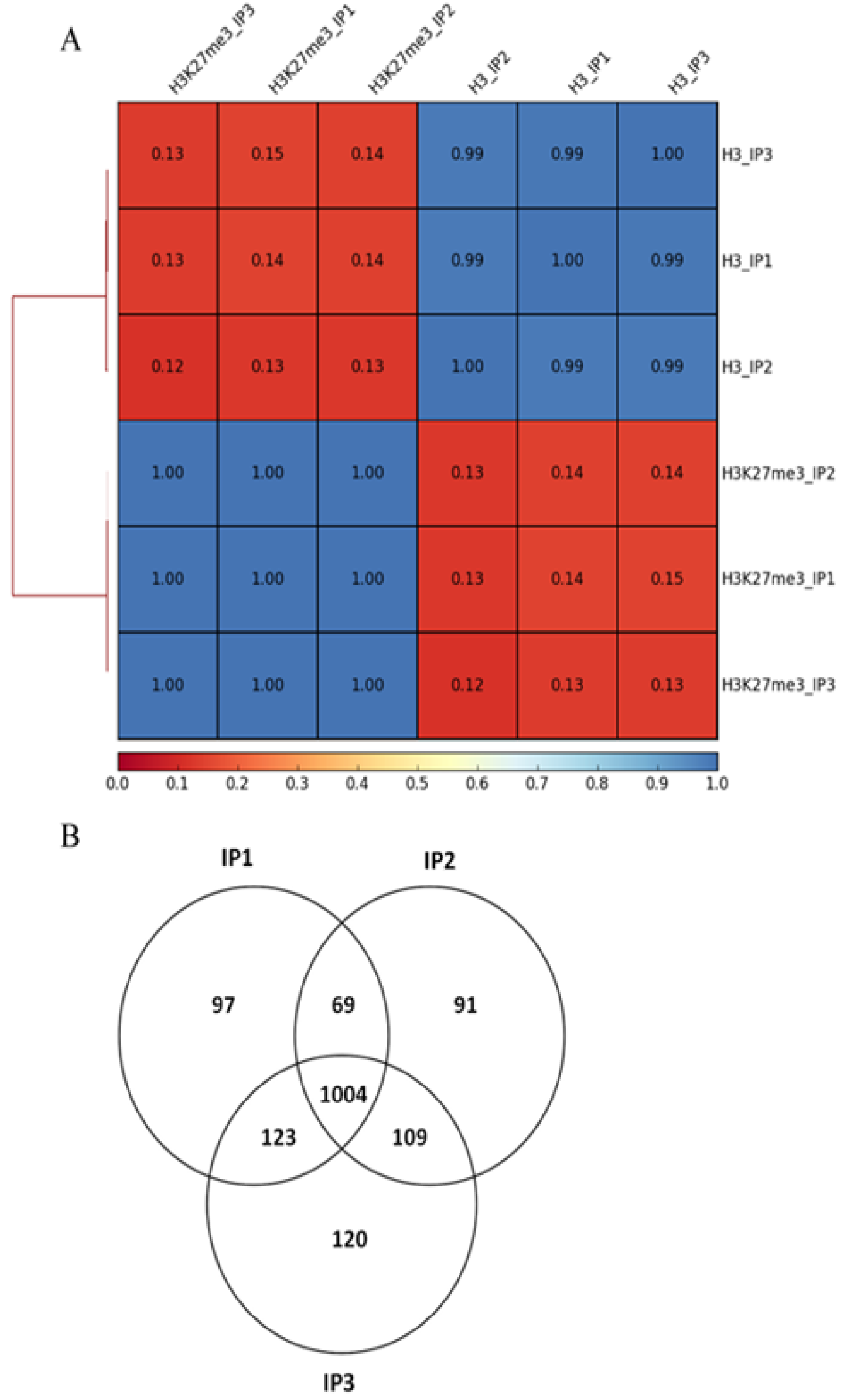

Figure S3. The quality of ChIP-seq data (A) Correlation for ChIP-seq experiment (replicates IP1-3). Values inside cells represent correlation coefficients calculated using Pearson method. Matrix for heatmap was computed in deepTools2 with bin size set to 1kb. Bins with zero and large counts were removed. Left side of the image shows hierarchical clustering between samples. (B) Correlation for RNA-seq experiment (replicates RNAseq_1-3). Matrix for scatterplots was generated in deepTools2 with bin size set to 0.2kb. Correlation was calculated using Pearson method. Bins with large counts were removed. X- and Y-axes show number of fragments in respective files. (B) An overlap of H3K27me3 peaks between biological replicates IP1-3.

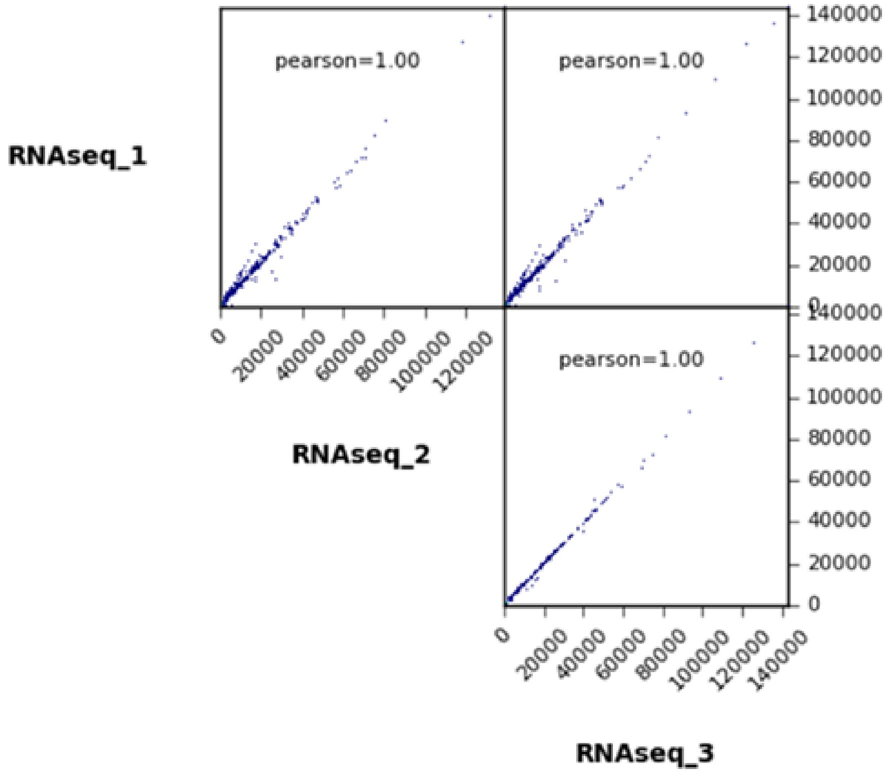

Figure S4. The quality of RNA-seq data. Correlation for RNA-seq experiment (replicates RNAseq_1-3). Matrix for scatterplots was generated in deepTools2 with bin size set to 0.2kb. Correlation was calculated using Pearson method. Bins with large counts were removed. X- and Y-axes show number of fragments in respective files.

Table S1. Distance between H3K27me3 peaks and gene locations.
Distance was obtained using 'closest' command from Bedtools v2.17 for 3 biological replicates (IP1-3) from chromatin immunoprecipitation experiment. Mean and median were calculated from distance values in base pairs for each peak. Peaks were divided into annotated ('ann.peak') and unannotated ('unannot.peak') subgroups. See main text for the details.

	IP1		IP2		IP3	
	ann. peak	unann. peak	ann. peak	unann. peak	ann. peak	unann. peak
Distance to annotated feature (mean)	542.9	543.8	539.2	520.5	504.1	514.0
Distance to annotated feature (median)	516.0	498.0	506.0	461.5	486.0	465.0

Table S2. Distance of H3K27me3 domain to the chromosome ends.

	distance [bp]			distance [bp]	
chromosome	to 5' end	to 3' end	chromosome	to 5' end	to 3' end
1	777	1321	11	144	44
2	19	247	12	126	28
3	3977	46	13	33	23
4	21	773	14	28	4734
5	41	453	15	18	18
6	216	24	16	27	3955
7	157	4756	17	74	36
8	149	31	18	236	22
9	22	28	19	97	987
10	1601	29	20	99	90
